# Testis‐Specific PDHA2 Is Required for Proper Meiotic Recombination and Chromosome Organisation During Spermatogenesis

**DOI:** 10.1111/cpr.70003

**Published:** 2025-02-20

**Authors:** Guoqiang Wang, Kailun Fang, Yongliang Shang, Xu Zhou, Qiqi Shao, Si Li, Ping Wang, Charlie Degui Chen, Liangran Zhang, Shunxin Wang

**Affiliations:** ^1^ Advanced Medical Research Institute Shandong University Jinan Shandong China; ^2^ State Key Laboratory of Molecular Biology, Shanghai Key Laboratory of Molecular Andrology, CAS Center for Excellence in Molecular Cell Science Chinese Academy of Sciences Shanghai China; ^3^ Center for Reproductive Medicine, State Key Laboratory of Reproductive Medicine and Offspring Health Shandong University Jinan Shandong China; ^4^ Center for Cell Structure and Function, Shandong Provincial Key Laboratory of Animal Resistance Biology, College of Life Sciences Shandong Normal University Jinan Shandong China; ^5^ Key Laboratory of Reproductive Endocrinology of Ministry of Education, National Research Center for Assisted Reproductive Technology and Reproductive Genetics Shandong University Jinan Shandong China; ^6^ Shandong Key Laboratory of Reproductive Medicine Shandong Provincial Clinical Research Center for Reproductive Health Jinan Shandong China

**Keywords:** chromosome structure, meiosis, PDHA2, recombination, spermatogenesis

## Abstract

Proper segregation of homologous chromosomes during meiosis requires crossovers that are tightly regulated by the chromosome structure. PDHA2 is the testis‐specific paralog of PDHA1, a core subunit of pyruvate dehydrogenase. However, its role during spermatogenesis is unclear. We show that PDHA2 knockout results in male infertility in mice, but meiotic DSBs in spermatocytes occur normally and are efficiently repaired. Detailed analysis reveals that mid/late recombination intermediates are moderately reduced, resulting in fewer crossovers and many chromosomes without a crossover. Furthermore, defective chromosome structure is observed, including aberrant histone modifications, defective chromosome ends, precocious release of REC8 from chromosomes and fragmented chromosome axes after pachytene. These defects contribute to the failure of pyruvate conversion to acetyl‐CoA, resulting in decreased acetyl‐CoA and precursors for metabolites and energy in the absence of PDHA2. These findings reveal the important functions of PDHA2 in ensuring proper crossover formation and in modulating chromosome structure during spermatogenesis.

## Graphical abstract

PDHA2, a testis‐specific subunit of pyruvate dehydrogenase, is required for the conversion of pyruvate to acetyl‐CoA. Its absence results in decreased acetyl‐CoA and precursors for metabolites and energy during spermatogenesis. This results in decreased histone acetylation, defective chromosome structure and moderately reduced crossovers, ultimately leading to male infertility.

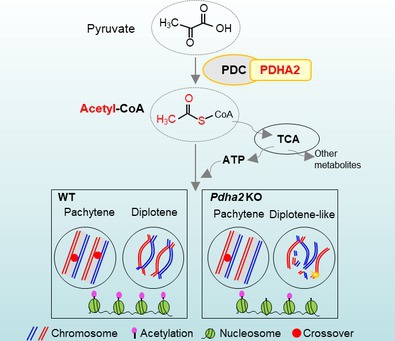

## 1 Introduction

Meiosis is a specialised type of cell division required to produce haploid gametes for sexual reproduction. During this process, one round of DNA replication is followed by two rounds of cell division: homologous chromosomes (homologues) from both parents and sister chromatids are segregated in the first and second rounds of cell division, respectively. Faithful segregation of homologues requires meiotic crossovers (COs), which link them together after disassembly of the synaptonemal complex (SC) [1, 2]. Failures in meiosis are the primary cause of infertility and congenital birth defects [3, 4].

Meiotic recombination is initiated from programmatically produced DNA double‐strand breaks (DSBs) catalysed by the SPO11 complex [5]. DSB ends are immediately processed to generate single‐stranded 3′ overhangs that are coated by replication protein A (RPA) and then replaced by RAD51/DMC1 recombinases [6, 7]. This coated ssDNA invades its homologous templates to form displacement loops (D‐loops) that are marked by MSH4/5 foci in *Sordaria*, mouse and human [8, 9, 10]. Most D‐loops develop into non‐crossovers [1, 11]. Only a small subset of D‐loops (~10% in mice) are selected to enter the CO pathway. During this process, D‐loops develop into stable recombination intermediates, single‐end invasions (SEIs), with strand synthesis. Double‐Holliday junctions (dHJs) then form when the second ends of DSBs are captured. Most, if not all, dHJs are specifically resolved to generate COs by the conserved MLH1–MLH3 nuclease, which also requires MutSγ, EXO1, RFC, PCNA and so forth [12, 13, 14, 15]. A set of ZMM proteins (Zip1‐4, Msh4/5 and Mer3 in budding yeast and their homologues in other organisms) elaborately ensure that these selected DSB sites are repaired to COs and that each pair of homologues obtains at least one CO [8, 11, 16, 17, 18, 19].

The pyruvate dehydrogenase complex (PDC) plays a critical role in energy metabolism by linking glycolysis to the oxidative tricarboxylic acid cycle. PDC consists of three enzymes: pyruvate dehydrogenase (E1), dihydrolipoamide acetyltransferase (E2) and dihydrolipoamide dehydrogenase (E3). They work sequentially to catalyse pyruvate to produce acetyl‐CoA [20]. PDC deficiency impairs mitochondrial metabolism and energy supply and is associated with a broader range of human diseases, including muscle hypotonia, mental retardation, seizures and lactic acidosis (e.g., [21]). The rate‐limiting enzyme E1 is a tetramer of two E1α and two E1β [22]. E1α has two isoforms, which are encoded by two different genes [23, 24, 25, 26]. The X‐linked PDHA1 is expressed in somatic cells and has 11 and 12 exons in mice and humans, respectively. PDHA2 is located on an autosome (chromosome 4 in humans and chromosome 3 in mice) with a single exon and is expressed only in the testes [23, 24, 25, 26]. The prevailing view is that PDHA2 is derived from PDHA1, given the high identity between their sequences [27]. However, the role of PDC in fertility is unclear, although studies suggest that mutations and/or altered expression of PDHA2 correlate with male infertility in both humans and hamsters [28, 29]. The testis‐specific expression of PDHA2 provides a unique opportunity to investigate the role of PDC in spermatogenesis.

In this study, we showed that PDHA2 substitutes the X‐linked PDHA1 for the conversion of pyruvate to acetyl‐CoA from mid/late prophase I of spermatogenesis. The consequence is the provision of sufficient ATP and metabolites for proper chromosome organisation and CO formation.

## 2 Materials and Methods

### 2.1 Mice

C57BL/6 mice were used in this study. Mice were housed under controlled environmental conditions with free access to water and food. The care protocols and use of mice in this study were reviewed and approved by the Animal Use Committee of Cheeloo College of Medicine, Shandong University.

The mouse *Pdha2* gene (GenBank: NM 008811.2) is located on chromosome 3 and contains a single exon. *Pdha2* knockout mice were generated by using the CRISPR/Cas9 system to delete a 13‐bp fragment (gggataaatccca) in the exon, resulting in a frameshift at the 108aa and the generation of a new stop codon at 128aa [30]. Mice were genotyped by PCR analysis of mouse tail DNA and DNA sequencing. PCR products were 561 and 574 bp for the mutant and WT, respectively. Sequences for the sgRNA and genotyping primers are listed in Table S1.

### 2.2 Antibodies

Guinea pig polyclonal antibody against mouse PDHA2 363–376aa (EDIANYLYHQDPPF), rabbit polyclonal antibody against mouse TEX11 788–946aa and guinea pig polyclonal antibody against mouse H1t 1–210aa were produced by Dai'an Biological Technology Incorporation (Wuhan, China); guinea pig polyclonal antibody against mouse HEI10 60–276aa and guinea pig polyclonal antibody against mouse RNF212 127–216aa were produced by ABclonal Biotechnology Co. Ltd. as previously described [31].

Primary antibodies were: guinea pig anti‐PDHA2 (WB, 1:1500; homemade), mouse anti‐SYCP3 (IF, 1:800; Abcam #ab97672), rabbit anti‐SYCP3 (IF, 1:800; Abcam #ab15093), rabbit anti‐SYCP1 (IF, 1:800; Abcam #ab15090), rabbit anti‐RPA2 (IF, 1:200; Abcam #ab76420), rabbit anti‐RAD51 (IF, 1:200; Thermo Fisher Scientific #PA5‐27195), rabbit anti‐DMC1 (IF, 1:200; Proteintech #13714‐1‐AP50), mouse anti‐phospho‐histone H2A.X (Ser139) (IF, 1:300; clone JBW301, Millipore #05‐636), mouse anti‐MLH1 (IF, 1:100; BD Biosciences #550838), rabbit anti‐CDK2 (IF, 1:200; Abcam #ab235941), mouse anti‐TRF1 (IF, 1:100; Abcam #ab10579), human anti‐ACA (IF, 1:200; Antibodies incorporated #15‐234‐0001), rabbit anti‐MSH4 (IF, 1:200; Abcam #ab58666), rabbit anti‐TEX11 (IF, 1:100), rabbit anti‐MZIP2 (1:200, kindly gift from Prof. Chao Yu, Zhejiang university), guinea pig anti‐RNF212 (IF, 1:100), guinea pig anti‐HEI10 (IF, 1:100), guinea pig anti‐H1t (IF, 1:100), pan acetylation monoclonal antibody (IF, 1:200; Proteintech #66289‐1‐Ig), rabbit anti‐histone H3 (acetyl K9) antibody (IF, 1: 200; WB, 1: 2000; Abcam #ab4441), rabbit anti‐phosphorylated histone H3 on Ser10 (IF, 1:200; Abcam #ab14955), rabbit anti‐H3K9me3 (IF, 1:200; WB, 1:2000; Abcam #ab176916), mouse FK2 antibody (IF, 1:200; WB, 1:2000; Ubiquigent #68‐0121‐500), rabbit anti‐histone H3 antibody (WB, 1:2000; Proteintech #17168‐1‐AP), rabbit anti‐REC8 antibody (IF, 1:200; WB, 1:1000; Affinity #DF4155), rabbit anti‐STAG3 antibody (IF, 1:200; Affinity #DF3470), mouse anti‐β‐actin (WB, 1:2000; Proteintech #66009‐1). Secondary antibodies were: HRP‐conjugated‐goat anti‐rabbit IgG (H+L) antibody (WB, 1:8000; Proteintech #SA00001‐2), HRP‐conjugated‐goat anti‐mouse IgG (H+L) antibody (WB, 1:8000; Proteintech #SA00001‐1), HRP‐conjugated‐goat anti‐guinea pig IgG (H+L) antibody (WB, 1:8000; Proteintech #SA00001‐12), CoraLite 594‐conjugated goat anti‐rabbit IgG (1:500, Proteintech #SA00013‐4), CoraLite 488‐conjugated goat‐rabbit IgG (1:500, Proteintech #SA00013‐2), CoraLite 594‐conjugated goat anti‐mouse IgG (1:500, Proteintech #SA00013‐3), CoraLite 488‐conjugated goat anti‐mouse IgG (1:500, Proteintech #SA00013‐1), TRITC‐conjugated donkey anti‐human IgG (1:500; Proteintech #SA00007‐11), Alexa Fluor 488‐conjugated goat anti‐guinea pig IgG H&L (1:800, Abcam #150185), Alexa Fluor 405‐conjugated goat anti‐guinea pig IgG H&L (1:800, Abcam #175678).

### 2.3 Reverse Transcription PCR and Quantitative Real‐Time PCR (qRT‐PCR)

Total RNA was isolated using TRIzol (#R701‐01, Vazyme, Nanjing, China), and cDNA was obtained from total RNA using the HiScript III 1st Strand cDNA Synthesis Kit (+ gDNA wiper) (#R312‐01, Vazyme, Nanjing, China) according to the manufacturer's instructions. PCR was performed at 95°C for 3 min, followed by 35 cycles of 95°C for 30 s, annealing at 60°C for 30 s, extension at 72°C for 1 min and a final extension at 72°C for 5 min. qRT‐PCR was performed with 2× Universal SYBR Green Fast qPCR mix (Abclonal, #RK21203) and gene‐specific primers using a CFX96 real‐time PCR detection system (Bio‐rad, USA). Relative mRNA levels (normalised to β‐actin expression) were determined using the comparative CT method [32]. Primers are listed in Table S1.

### 2.4 Protein Extraction and Western Blot

Protein lysates were prepared from testes or spermatocytes collected from 8‐week‐old male mice using RIPA buffer (ThermoFisher Scientific, Grand Island, NY; #89900) supplemented with a protease inhibitor cocktail (CWBio, Beijing, China; #CW2200). Protein concentration was determined by the BCA method according to the manufacturer's instructions (Vazyme, Nanjing, China; #E112‐01). Proteins were separated on a 10% SDS‐polyacrylamide gel and transferred to nitrocellulose (NC) filter membranes (Millipore, Billerica, MA). After blocking, the membranes were incubated with the appropriate primary antibody overnight at 4°C and then with the appropriate HRP (horseradish peroxidase)‐conjugated secondary antibody for 2 h at room temperature. Membranes were exposed to the Super Signal West Dura detection solution (ThermoFisher Scientific, Grand Island, NY; #34076) according to the manufacturer's instructions and imaged using a Tianneng Chemiluminescence Imaging Station.

### 2.5 Fertility Test and Sperm Count

Fertility test and sperm count were performed as previously described [31]. For the male fertility test, each male mouse was caged with two 6‐ to 8‐week‐old WT female mice, and vaginal plugs were checked every morning. Once a vaginal plug was identified (Day 1 postcoitus), the male was allowed to rest for 2 days, and then, for another round of mating, the plugged female was separated and caged alone, and the pregnancy was recorded. The number of newborn pups per litter was recorded. Female fertility was assessed in a similar manner, except that each female was caged with one WT male mouse every time. For sperm count, 8‐week‐old mice were sacrificed by cervical dislocation, and the cauda epididymis was immediately transferred to 1 mL of prewarmed PBS. Then, the cauda epididymis was cut into pieces with scissors and incubated in a 37°C water bath for 15 min to fully release sperm. The sperm number was counted using a haemocytometer under a light microscope (B203, Cnoptec, Chongqing, China).

### 2.6 Histological Analysis and Immunostaining

Histological analysis and immunostaining of testis and epididymis sections were performed as previously described [33]. Mice testes and cauda epididymis were isolated immediately after euthanasia, fixed in Bouin's solution (Sigma, #HT10132‐1L) or 4% PFA (Servicebio, #G1101) for 24 h, and then embedded in paraffin. Five micrometre sections were cut and mounted to microscope slides. After deparaffinisation, slides were stained with haematoxylin and eosin (HE) or appropriate antibodies.

### 2.7 TdT‐Mediated dUTP Nick‐End Labelling (TUNEL) Assay and Periodic Acid Schiff (PAS) Assay

The TUNEL assay was prepared as we previously described [33]. Briefly, testis sections were deparaffinised in xylene, rehydrated in a graded ethanol series (100%, 95%, 90%, 80%, 70%, 50% ethanol and sterile water), and permeabilised with proteinase K (20 mg/mL) in 10 mM Tris‐HCl (pH 7.5) for 15 min at room temperature. After blocking with 3% H_2_O_2_ in 1× PBS for 20 min at room temperature, TUNEL reagent mix (KeyGEN Biotech, China; #KGA7072) was applied, and the slides were incubated at 37°C for 1 h according to the manufacturer's protocol.

PAS staining was performed using the Periodic Acid Schiff (PAS) Stain Kit (KeyGEN Biotech, China; #KGA222‐1). Stages of the seminiferous epithelium cycle and spermatid development were determined as previously described [31].

### 2.8 Chromosome Spread and Immunostaining

Chromosome spread and subsequent immunostaining were performed as previously described [33]. Briefly, seminiferous tubules were incubated in hypotonic extraction buffer (30 mM Tris, 50 mM sucrose, 17 mM trisodium citrate dihydrate, 5 mM EDTA, 0.5 mM dithiothreitol (DTT) and 0.5 mM phenylmethylsulphonyl fluoride (PMSF), pH 8.2) at room temperature for 30 min. Cells were then suspended in 100 mM sucrose and spread on slides in 1% PFA (pH 9.2) containing 0.1% Triton X‐100. The slides were dried in a humidified chamber for at least 3 h. For immunostaining, slides were washed, blocked and incubated with the appropriate primary antibody at 4°C overnight. After washing, the slides were incubated with the appropriate secondary antibody at room temperature for 2 h. After drying, an anti‐fade solution (Solarbio, S2110/S2100) was applied. Images were visualised and acquired using an epifluorescence microscope (BX52, Olympus).

### 2.9 Okadaic Acid (OA) Treatment

Short‐term culture of testicular cells and OA treatment were carried out as previously reported [33]. In brief, freshly dissected testes of 8‐week‐old male mice were decapsulated. The seminiferous tubules were rinsed and digested in 1× Krebs buffer (120 mM NaCl, 4.8 mM KCl, 25.2 mM NaHCO_3_, 1.2 mM KH_2_PO_4_, 1.2 mM MgSO_4_, 1.3 mM CaCl_2_ and 11.1 mM dextrose) containing collagenase I (1.6 mg/mL). The cell suspension was passed through a 100 μm nylon mesh. ~2.5 × 10^6^ testicular cells were incubated overnight in α‐MEM medium containing 5% streptomycin, 7.5% penicillin, 0.29% Dl‐lactic acid sodium salt, 0.59% HEPES and 5% foetal bovine serum, treated with 4 μM OA (Cell Signalling Technology, #5934S) for 5 h, and then spread on slides. After air‐drying, the slides were stained with Giemsa (KeyGEN BioTech, China; #KGA228).

### 2.10 Purification of Pachytene Spermatocytes

Pachytene spermatocytes were isolated and collected by velocity sedimentation at unit gravity (STA‐PUT) as previously described [34]. Briefly, testes were harvested, decapsulated and digested into a single cell suspension. After filtering through a 40 μm nylon cell strainer (Falcon, BD, NJ, United States; #352340), cells were resuspended in 10 mL DMEM/F12 medium containing 0.5% BSA and loaded into a cell separation apparatus (BOMEX Corporate) containing a 2% ~ 4% BSA gradient in 600 mL DMEM/F12 medium. After 3 h of sedimentation, the cells were collected in a series of tubes. The purity and stages of spermatocytes were examined by immunostaining the axis component SYCP3 and the SC component SYCP1. Typically, spermatocytes with a diameter of 12–18 μm are at pachytene.

### 2.11 Quantification of Mitochondrial DNA (mtDNA)

Total DNA was extracted from purified pachytene spermatocytes using the lysis buffer (1 M (NH_4_)_2_SO_4_ 16.6 mL, 1 M Tris‐HCl 66.9 mL, 1 M MgCl_2_ 6.7 mL, β‐mercaptoethanol 350 μL, 0.5 M EDTA (pH 8.0) 134 μL, ddH_2_O 9.8 mL). Relative mtDNA copies were determined by qPCR using mitochondrial 16S‐specific primers. The nuclear apolipoprotein B (*ApoB*) served as a reference. The PCR reaction was performed with an initial denaturation at 95°C for 3 min followed by 40 cycles of denaturation at 95°C for 15 s, annealing at 60°C for 30 s, extension at 72°C for 30 s and a final extension at 72°C for 5 min. Primers are listed in Table S1.

### 2.12 Determination of the ATP Level

ATP levels were determined using the Enhanced ATP Assay Kit (Beyotime Institute of Biotechnology, Nanjing, China; #S0027) according to the manufacturer's instructions. In brief, the entire testicular tissue was homogenised, cells were lysed and a luciferase mixture was added. Luminescence was examined and compared with the standard curve.

### 2.13 Determination of Pyruvate Content

Pyruvate concentration was determined with a pyruvate detection kit (#A081, Nanjing Jiancheng Bioengineering Institute) using the NADH oxidase method according to the manufacturer's instructions. Spectrophotometry at 505 nm was measured.

### 2.14 Measurement of Acetyl‐CoA

Acetyl‐CoA levels in protein‐free mouse testis lysates were determined using an assay kit (Jining Shiye, China; #JN20555) according to the manufacturer's instructions. Testes were homogenised in pre‐cooled PBS and centrifuged. The supernatant and the standard sample were added to different wells of the microplate coated with mouse anti‐acetyl‐CoA antibodies. Then, the HRP‐conjugated anti‐mouse IgG antibody was added to each well and incubated at 37°C for 1 h. After washing, TMB (3,3′,5,5′‐tetramethylbenzidine) substrate colorimetric solution (A and B) was added and incubated for 15 min in the dark. The reaction was stopped with H_2_SO_4_. The OD value was measured at a wavelength of 450 nm. The protein concentration of the supernatant was determined by the BCA assay, and the concentration of acetyl‐CoA was shown as picomoles of acetyl‐CoA per gram of protein (pmol/g).

### 2.15 RNA Sequencing (RNA‐Seq) and Data Analysis

RNA‐seq was performed at Novogene Corporation Inc. Total RNA was extracted from purified pachytene spermatocytes using a TRIzol reagent (CWBIO, CW0580) according to the manufacturer's instructions. The RNA quality and quantity were evaluated using the RNA Nano 6000 Assay Kit of the Bioanalyzer 2100 system (Agilent Technologies, USA). Sequencing was performed on an Illumina HiSeq PE150 (Novogene Corporation Inc.). DESeq2 R package (1.20.0) was used to analyse the differentially expressed genes with the standard threshold of ‘*p*_adj_ < 0.05 and |log2(foldchange)| > 1’ [35, 36]. Gene Ontology (GO) analysis of differentially expressed genes was performed using ClusterProfiler.

### 2.16 ATAC‐Seq and Data Analysis

ATAC‐seq (assay for transposase accessible chromatin using sequencing) was performed as previously described [33]. In brief, nuclei from 8‐week mice pachytene spermatocytes were resuspended in the Tn5 transposase reaction mix and incubated for 30 min at 37°C. The transposed DNA fragments were purified using the MinElute PCR Purification Kit (Qiagen, Beijing, China), amplified using 1× NEBNext High‐Fidelity PCR Master Mix (New England Biolabs, MA, USA), and then purified using MinElute PCR Purification Kit (Qiagen, Beijing, China). The samples were sequenced as 150 bp paired‐end reads on the Illumina Novaseq6000 instrument by Jiayin Biotechnology Institute (Shanghai, China).

After adaptor sequence removal, the reads were aligned to the mouse reference genome (Release mm10) using BWA [37]. Peak calling was performed using MACS2 [38]. Each peak region extended 200 bp from the 5′ end to the 3′ end to extract the DNA sequence. BEDTools was used to annotate peaks. Deeptools software was used to analyse the signal distribution near the gene. DESeq2 was used to identify differential accessible peaks with cut‐off |log2 fold change| ≥ 1 and *p* < 0.05. GO was used to elucidate the biological processes (BPs) of genes associated with the accessible chromatin regions.

### 2.17 Acetylome Analysis by LC–MS/MS

The proteomic and acetylome analyses were performed at Jingjie PTM Biolab (Hangzhou, China) Co. Ltd. with pachytene spermatocytes from 8‐week mice. After protein extraction and trypsin digestion, acetylated peptides were enriched with anti‐acetyl lysine beads (Cat#PTM‐104, PTM Bio, Hangzhou, China). The acetylated peptides were identified by label‐free liquid chromatography–tandem mass spectrometry (LC–MS/MS) analysis. Mass spectrometry data were searched against a Swissprot protein sequence database (Mus_musculus_10090_SP_20201214.fasta) by Maxquant (v1.6.15.0). False discovery rate (FDR) of protein, peptide and PSM was adjusted to < 1%. Differentially acetylated sites were identified with the standard threshold of ‘*p* < 0.05 and fold change > 1.5’. GO analysis was performed using DAVID database.

### 2.18 Quantification and Statistical Analysis

Statistical analysis was performed with GraphPad Prism 7.01 software. Data were presented as mean ± error bars that represent the standard error of the mean (SEM), standard deviation (SD) or 95% confidence interval, as indicated in the figure legends. Sample sizes were described in figure legends. Student's *t*‐test was used to determine the statistical significance of the number of events between two groups of samples. One‐way analysis of variance (ANOVA) followed by Dunn's test was performed for statistical comparison between three or more groups of samples. Chi‐square was used to determine the statistical significance of frequencies between samples. Significance levels are stated: n.s. (not significant), *p* ≥ 0.05; *p < 0.05; **p < 0.01; ***p < 0.001.

## 3 Results

### 3.1 PDHA2 Is Preferentially Expressed in Testes and Is Required for Male Fertility

**FIGURE 1:**
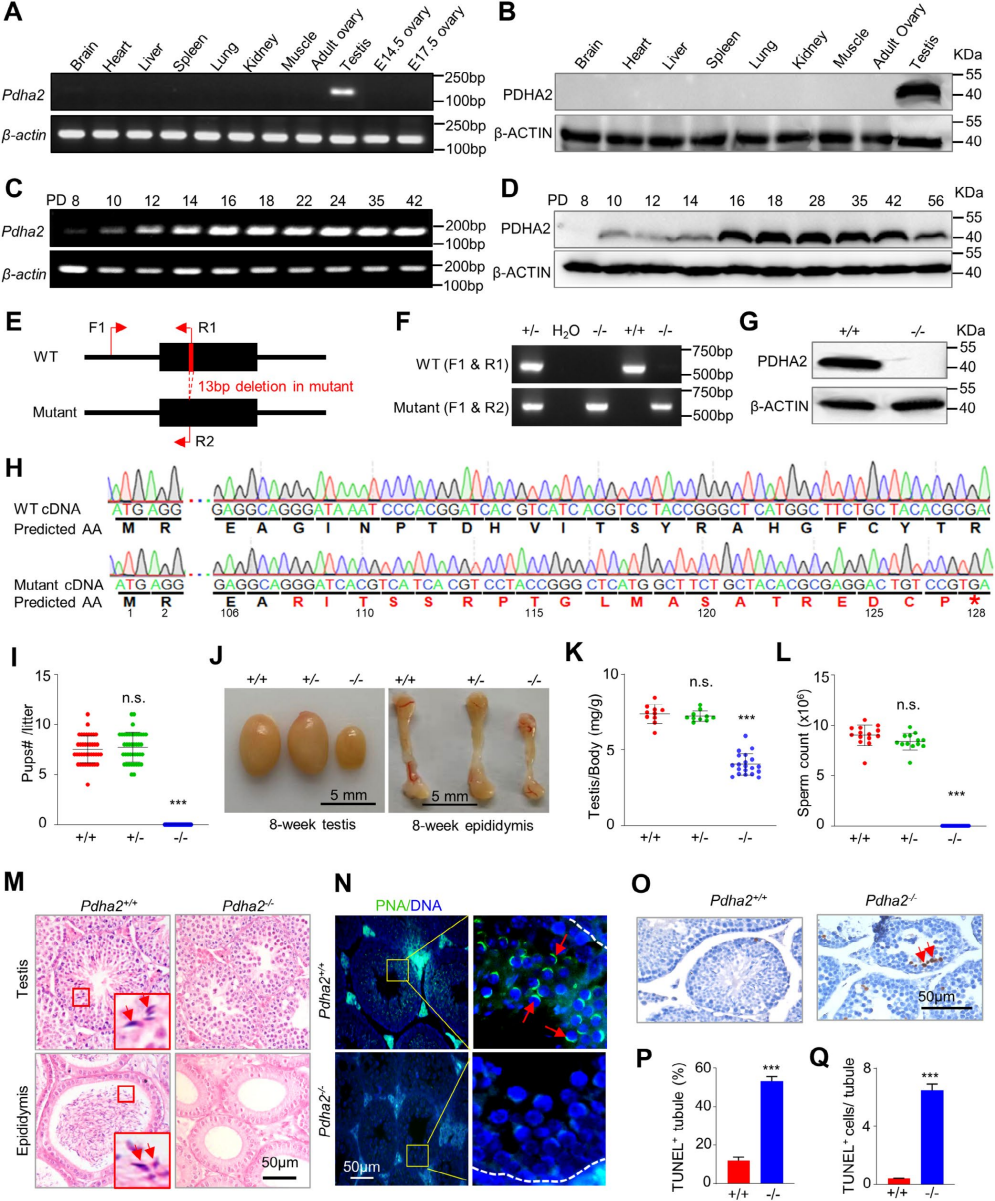
PDHA2 is preferentially expressed in testes and is required for male fertility. (A and B) RT‐PCR (A) and western blot (B) to examine *Pdha2* mRNA and protein abundance in various mouse tissues, respectively. (C and D) RT‐PCR (C) and western blot (D) to detect *Pdha2* mRNA and protein in mouse testes at different stages, respectively. β‐actin served as the control. (E) Schematic diagram to show the gene structure of *Pdha2* and the mutant allele with a 13‐bp deletion in the single exon. Primers used for genotyping were indicated: F1 matches with both WT and the mutant sequence, R1 only matches with the WT sequence and R2 only matches with the mutant sequence. (F) Genotyping of WT and *Pdha2* knockout mice by PCR with primer pairs F1/R1 and F1/R2 and templates of mouse toe lysate. H_2_O was used as the blank control. (G) Western blot to detect PDHA2 protein from 8‐week‐old mouse testes. β‐actin served as a control. (H) Sequences of the RT‐PCR product from WT and mutant testes. The asterisk indicates the new stop codon in the mutant. (I) *Pdha2* ^*−/−*^ male mice were sterile. Each dot represents the number of pups per litter. *n* = 41, 42 and 41 from left to right (six male mice for each genotype). (J) *Pdha2* ^*−/−*^ mice have smaller testes (left) and epididymides (right) *Pdha2* ^*+/+*^ and *Pdha2* ^*+/−*^mice at 8 weeks old. (K) Quantification of testis/body weight. *n* = 10, 10 and 20 mice from left to right). (L) Determination of sperm counts collected from caudal epididymides. *n* = 14, 13 and 18 8‐week‐old mice from left to right. (M) HE staining of histological sections of testes (top row) and caudal epididymides (bottom row) from 8‐week‐old mice. Enlargements of selected areas with arrows to show sperm. (N) Immunostaining of 8‐week‐old testis sections with an antibody against PNA (peanut agglutinin; red arrowheads) and the enlargement of selected regions on the right. Nuclei were counterstained with DAPI. (O) Representative images to show TUNEL^+^ spermatocytes from testis sections (red arrows). (P and Q) Quantification of TUNEL‐positive seminiferous tubules (P) and TUNEL‐positive cells per tubule (Q) from three independent experiments. Error bar, SD (I, K and L) or SEM (P and Q); n.s. (not significant) *p* > =0.05; ***p < 0.001; two‐tailed *t*‐test (I, K, L, P and Q).

Proteomic analysis of mouse germ cells identified PDHA2, the catalytic subunit of the PDC, as a new candidate regulating meiosis [25, 30, 39]. In mice, this gene is 22.35 kb and located on chromosome 3, has a single exon with an open reading frame of 1173 nucleotides, and encodes a protein with 391 amino acid residues. Sequence analysis revealed that PDHA2 exists only in vertebrates and is highly conserved (Figure S1). As previously reported [24, 26, 28, 39], PDHA2 was expressed only in testes at both mRNA and protein levels in mice and as in humans (Figure 1A,B). Its expression was detectable from postnatal day 8 (PD8) testes, gradually increased, and was abundant from PD16, when more than half of the spermatocytes are at pachytene (Figure 1C,D). Several examples of the testis‐specific expression of an autosomal retrogene to substitute its X‐linked paralog (which would be silenced) during spermatogenesis have been reported [40].

To explore the roles of PDHA2 in meiosis, the *Pdha2* KO mice were generated using the CRISPR/Cas 9 technology to delete a 13 bp fragment in the single exon (Figure 1E). The KO was confirmed by genotyping PCR combined with DNA sequencing and western blot (Figure 1F–H). *Pdha2* ^*−/−*^ mice were viable and appeared to develop normally. *Pdha2* ^*−/−*^ female mice were fertile, and the ratio of ovary/body weight and number of pups per litter were also comparable to WT (Figure S2). However, *Pdha2* ^*−/−*^ male mice were completely infertile (Figure 1I). The sizes of testes and epididymis of *Pdha2* ^*−/−*^ mice were smaller than those of 8‐week‐old WT mice (Figure 1J,K). Further investigation showed that the cauda epididymis of WT was full of mature spermatozoa, whereas, none was observed in the mutant (Figure 1L). This result was confirmed by H&E staining of cauda epididymis sections (Figure 1M). H&E staining of testis sections also showed no post‐meiotic spermatids in the seminiferous tubules (Figure 1M). The absence of spermatids was further confirmed by the absence of peanut agglutinin (PNA) signal, an acrosomal marker (Figure 1N). Moreover, more TUNEL^+^ spermatocytes were observed in *Pdha2* ^*−/−*^ than in WT testis sections (Figure 1O–Q). These results suggest that PDHA2 is essential for spermatogenesis and male fertility, and its absence led to spermatocyte elimination prior to the formation of post‐meiotic spermatids.

### 3.2 PDHA2 Is Required for Proper Meiosis Progression

**FIGURE 2:**
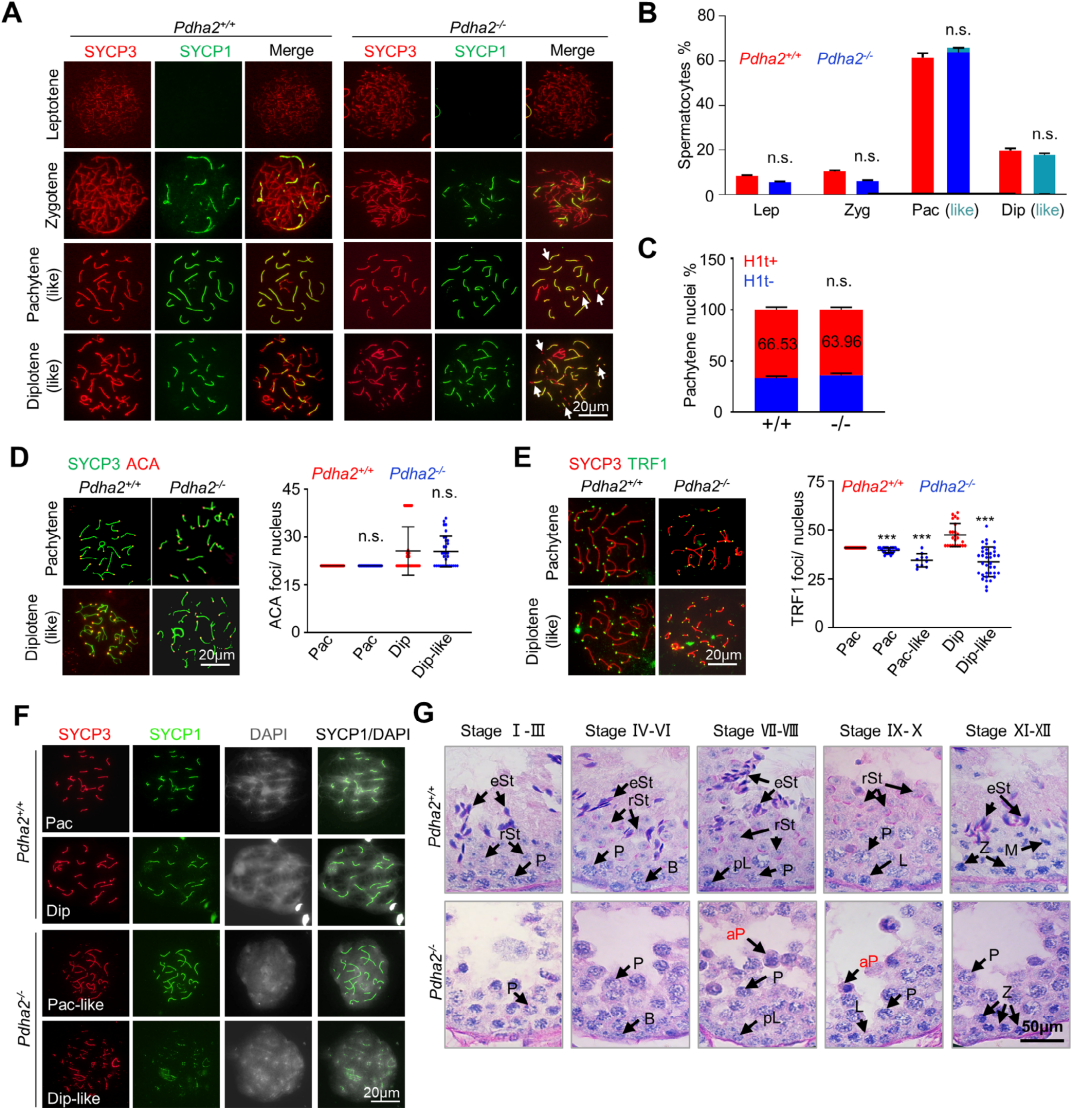
PDHA2 is required for proper meiotic progression. (A) Immunostaining of SYCP3 (red) and SYCP1 (green) in surface spread spermatocytes. Note fragmented axes (arrows) in pachytene‐like and diplotene‐like spermatocytes. (B) Percentages of spermatocytes at different substages of meiotic prophase I at postnatal day 20. *n* = 1413 (*Pdha2* ^*+/+*^) and 1463 (*Pdha2* ^*−/−*^) spermatocytes. Dip, diplotene; Lep, leptotene; Pac, pachytene; Zyg, zygotene. (C) Quantification of percentages of H1t+ and H1t− pachytene spermatocytes. *n* = 861 (*Pdha2* ^*+/+*^) and 958 (*Pdha2* ^*−/−*^) pachytene nuclei. (D and E) Immunostaining surface spread spermatocytes for ACA (D) and TRF1 (E) and quantification. From left to right: *N* = 67, 58, 52 and 32 (D); 45, 24, 10, 24 and 36 (E) nuclei. (F) DAPI staining to show chromatin morphology. (G) Periodic acid Schiff (PAS) staining of 8‐week‐old testis sections. aP, apoptotic pachytene; B, B‐type spermatogonium; Dip, diplotene; M, metaphase I; L, leptotene; P, pachytene; pL, pre‐leptotene; rSt, round spermatid; St, elongate spermatid; Z, zygotene. Error bar, 95% confidence interval (B and C) or SD (D and E). ns (not significant) *p* ≥ 0.05; ***p < 0.001; Chi‐square test (B and C) or two‐tailed *t*‐test (E and F).

The percentages of spermatocytes at different stages from leptotene to diplotene reflect the progression of meiosis through prophase I, which can be reliably distinguished based on chromosome morphologies and homologue synapsis status [31, 41]. PD20 spermatocytes only have the first wave of synchronised spermatocytes. Leptotene is defined by the presence of short stretches of SYCP3 (a homologue axis component), indicating developing chromosome axes, but no SYCP1 (a central SC element) (Figure 2A, top row). Zygotene is defined by continuous SYCP3 along the chromosome axes and stretches of SYCP1 indicating the partial homologue synapsis (Figure 2A, second row). When all autosomes are fully synapsed, as indicated by continuous SYCP1 staining, cells enter pachytene (Figure 2A, third row). After that, SYCP1 is progressively disassembled, but intact SYCP3 axes are maintained, indicating cells progress to diplotene (Figure 2A, bottom row). At late diplotene, homologues are only connected at chiasma sites.

Comparable percentages of spermatocytes (PD20 testes) at each stage from leptotene to pachytene were observed between the mutant and WT, except that a few (~2%) abnormal/pachytene‐like spermatocytes with fragmented axes were also observed in the mutant (Figure 2A,B). There were ~20% diplotene spermatocytes in the WT, but typical diplotene spermatocytes were not observed in the mutant. Instead, there was a comparable fraction (~21%) of spermatocytes with fragmented axes (judged by SYCP3) and partial SC (judged by SYCP1) in the mutant, which was never seen in WT (Figure 2A,B). These abnormal spermatocytes were considered diplotene‐like cells because it looked like SYCP3 was immediately disassembled from the axis fragments where SYCP1 marked SC was disassembled: SYCP3 was still visible in regions without SYCP1 in a small fraction (1.7%) of these spermatocytes. This assessment was further supported by the following observations. (1) The mutant and WT had similar fractions of early and mid/late pachytene spermatocytes (by H1t staining), respectively, indicating that *Pdha2* ^*−/−*^ had morphologically normal pachytene spermatocytes (Figure 2C). (2) These abnormal spermatocytes showed centromeric signals similar to WT when examined with a human anti‐centromere antibody (ACA) (Figure 2D). However, mutant pachytene spermatocytes had fewer TRF1 (a component of the shelterin complex) foci, indicating defective chromosome ends. Fewer TRF1 foci were also observed in diplotene‐like spermatocytes (Figure 2E). (3) The more diffuse DAPI signal suggests defective chromosome axes in abnormal spermatocytes (Figure 2F). These results suggest that PDHA2 deletion results in defective diplotene chromosome morphology: fragmented chromosome axes at desynapsed regions.

Meiotic defects around the diplotene in *Pdha2* KO mice were further confirmed by PAS staining of testis sections. In WT seminiferous tubules, spermatocytes from leptotene to metaphase I and round and elongating spermatids are well organised (Figure 2G, top row). The seminiferous epithelium can be divided into 12 stages (Stages I–XII) according to the development of spermatocytes and spermatids [41]. In *Pdha2* ^*−/−*^ testes, spermatids were never found, and the stages of the seminiferous epithelium were identified based on the types of spermatocytes (Figure 2G). In addition, many pachytene/diplotene‐like spermatocytes with abnormally condensed nuclei of Stage VII appeared in *Pdha2* ^*−/−*^ mice [41] (Figure 2G; ‘aP’). These results suggest that defective *Pdha2* ^*−/−*^ spermatocytes are likely to be eliminated by apoptosis, resulting in male infertility.

Spermatocytes from pachytene to metaphase I are easily recognised based on chromosome condensation and morphology by Giemsa staining. Pachytene chromosomes are less condensed, diplotene chromosomes are linear, and chromosomes at prometaphase I are more condensed with visible chiasmata. At metaphase I, condensed bivalents linked at chiasmata can be clearly distinguished (Figure S3A, top row). In PD20 testes, ~62%, ~23%, ~13% and ~2% of spermatocytes were at pachytene, diplotene, prometaphase I and metaphase I, respectively (Figure S3B). However, only ~84% pachytene and ~16% diplotene spermatocytes were observed in the mutant (Figure S3A,B). Consistently, the signal of phosphorylation of histone H3 at Ser^10^ (pHH3), an indicator of chromatin condensation from late diplotene onward, was absent in the mutant (Figure S3C,D; [42]). OA, a protein phosphatase inhibitor, can be used in vitro to induce mid‐pachytene or later spermatocytes to enter MI prematurely [43]. When spermatocytes were treated with OA, ~80% of WT spermatocytes were observed at MI, but no MI spermatocytes were observed in the mutant and only ~17% were at diplotene (Figure S3B). These results suggest that the mutant has defective cell cycle progression and the morphologically normal pachytene spermatocytes actually have defective chromosome organisation.

### 3.3 PDHA2 Is Required for Efficient Crossover Formation

**FIGURE 3:**
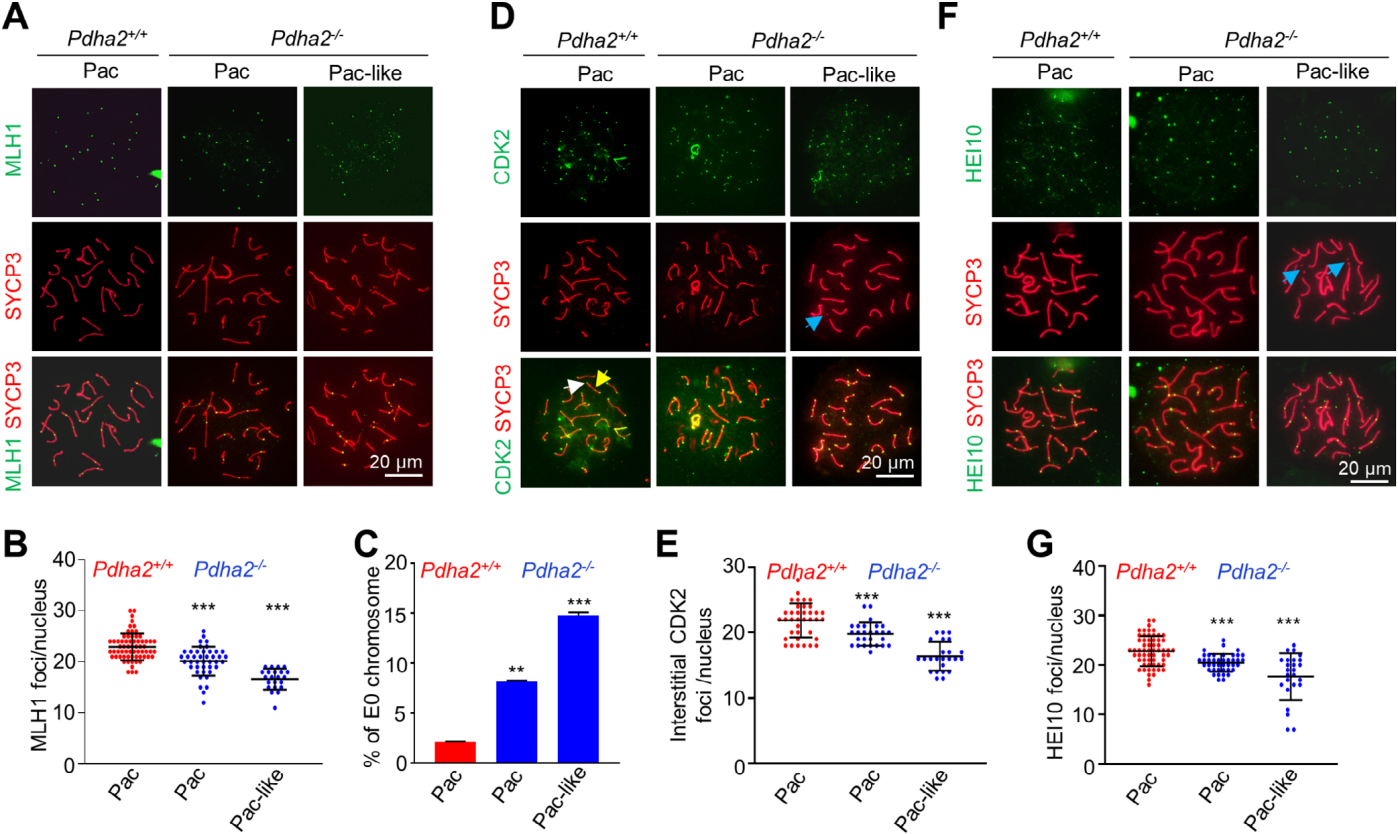
PDHA2 is required for proper crossover formation. (A) Representative images to show MLH1 (green) in surfaced spread spermatocytes. (B) Quantification of MLH1 foci in (A). From left to right, *n* = 65, 42 and 22 nuclei. (C) Percentages of pachytene chromosomes without MLH1 focus (E0 chromosomes). From left to right, *n* = 192, 166 and 63 nuclei. (D) Representative images to show CDK2 (green) on surfaced spread spermatocytes. White arrow, a terminal CDK2 focus; yellow arrow, an interstitial CDK2 focus; blue arrow, fragmented axes. (E) Quantification of CDK2 foci in (D). From left to right, *n* = 37, 27 and 24 nuclei. (F) Representative images to show HEI10 (green) on surfaced spread spermatocytes. Blue arrow, fragmented axes. (G) Quantification of HEI10 foci in (F). From left to right, *n* = 60, 41 and 25 nuclei. Error bar, SD (B, E and G) or 95% confidence interval (C). **p < 0.01; ***p < 0.001; ANOVA followed by Dunn's test. Pac, pachytene; Pac‐like, pachytene‐like.

One hallmark of meiosis is the meiotic recombination process that results in the formation of COs. In mice, ~90% of COs are marked by the late recombination nodule component MLH1 [17, 44]. As reported, ~23 MLH1 foci were observed in each WT pachytene spermatocyte; however, the number was slightly but significantly decreased to ~20 even in morphologically normal pachytene spermatocytes and to ~17 in pachytene‐like spermatocytes in *Pdha2* KO mice (Figure 3A,B). Correspondingly, the frequency of spermatocytes with no‐MLH1 autosomes was largely increased (Figure 3C). COs can also be marked by the ubiquitin ligase HEI10 and interstitially localised cyclin‐dependent kinase CDK2 foci at pachytene [17, 44]. When examined, each pachytene spermatocyte showed an average reduction of 2 foci for both types of foci in the mutant (20 vs. 22 for CDK2 and 21 vs. 23 for HEI10; Figure 3D–G). Thus, PDHA2 is required for efficient CO formation.

To further understand the role of PDHA2 in CO formation, the meiotic recombination process was examined in detail. Meiotic CO recombination initiates from programmed DSBs, which can be marked by phosphorylated H2AX (γH2AX) [45]. In WT, abundant γH2AX appears in leptotene and zygotene spermatocytes and gradually decreases and is barely observed on autosomes at pachytene, meanwhile accumulating on XY chromosomes (XY body) (Figure S4A,B). Similar dynamics of γH2AX signal were observed in *Pdha2* ^*−/−*^ from leptotene to pachytene (Figure S4A–C). However, in *Pdha2* ^*−/−*^ diplotene‐like spermatocytes, an apparent γH2AX signal reappeared on autosomes again (Figure S4A–C). Resected ssDNA ends of DSBs are coated by RPA, which is then replaced by RAD51 and DMC1 to promote D‐loop formation [6, 7, 46, 47]. In both WT and mutant spermatocytes, RPA, DMC1 and RAD51 all showed very similar numbers of foci and dynamics (Figure S4D–I). These results indicate meiotic DSBs occur and are processed normally in the absence of PDHA2, although additional DSBs or apoptosis may occur in diplotene‐like spermatocytes in the mutant.

**FIGURE 4:**
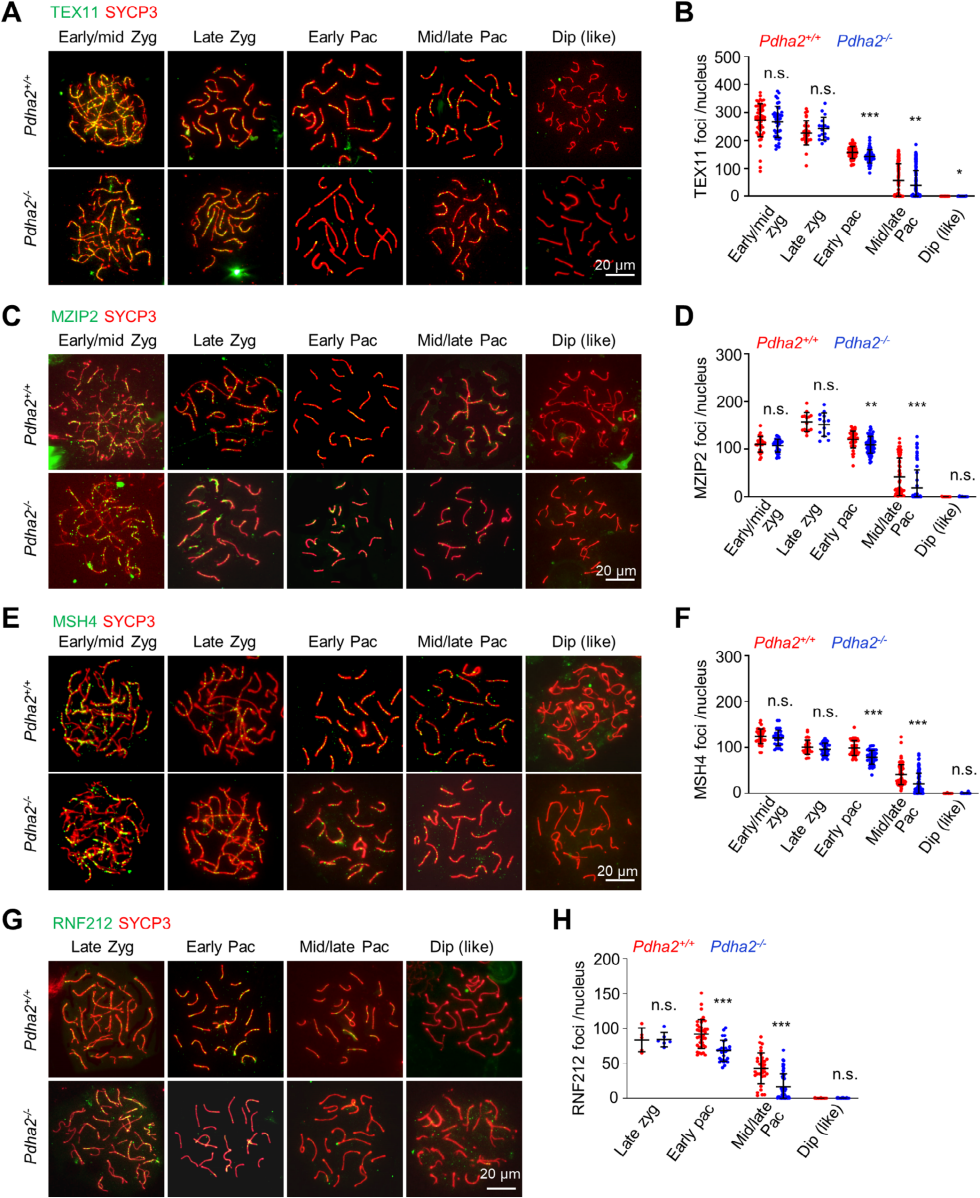
PDHA2 is required to stabilise mid/late recombination intermediates. (A and B) Representative images to show TEX11 in surface spread spermatocytes (A) and quantification (B). From left to right, *n* = 59, 40, 31, 17, 66, 63, 109, 196, 26 and 29 nuclei. (C and D) Representative images to show MZIP2 in surface spread spermatocytes (C) and quantification (D). From left to right, *n* = 27, 33, 14, 13, 42, 58, 65, 83, 34 and 15 nuclei. (E and F) Representative images to show MSH4 in surface spread spermatocytes (E) and quantification (F). From left to right, *n* = 38, 32, 32, 40, 51, 45, 98, 81, 42 and 23 nuclei. (G and H) Representative images to show RNF212 in surface spread spermatocytes (G) and quantification (H). From left to right, *n* = 5, 6, 39, 27, 33, 55, 35 and 35 nuclei. Error bar, SD (B, D, F and H). n.s. (not significant) *p* > =0.05; *p < 0.05; **p < 0.01, ***p < 0.001; two‐tailed *t*‐test. Dip, diplotene; Dip‐like, diplotene‐like; Early/mid Zgy, early/middle zygotene; Early Pac, early pachytene; Late Zyg, late zygotene; Mid/late Pac, middle/late pachytene.

The ZZS complex (Zip2‐Zip4‐Spo16 in yeast and MZIP2‐TEX11‐SPO16 in mouse) stabilises early recombination intermediates for the efficient recruitment of MSH4–MSH5, which further promotes and stabilises late recombination intermediates [48, 49, 50, 51, 52, 53]. When examined, comparable numbers of TEX11, MZIP2 and MSH4 foci were detected in both *Pdha2* ^*−/−*^ and WT zygotene spermatocytes (Figure 4A–F). Along with meiosis progression, the number of these foci decreased more rapidly, and thus there were significantly fewer foci in *Pdha2* ^*−/−*^ pachytene spermatocytes than in WT (Figure 4A–F). The SUMO ligase RNF212 stabilises MSH4–MSH5 and ZZS complexes to promote CO formation [16, 17, 18]. Comparable numbers of RNF212 foci appeared on synapsed chromosome regions at late zygotene in both WT and the mutant (Figure 4G,H). However, from early pachytene, fewer RNF212 foci were observed in the mutant than in WT (68 vs. 92 at early pachytene; 16 vs. 43 at mid/late pachytene; Figure 4G,H). These and the above analyses suggest that the formation of homologous recombination intermediates is not affected, but they are less stabilised and thus released more rapidly in *Pdha2* ^*−/−*^ spermatocytes. As a result, a small fraction of them should be stabilised and developed into COs at pachytene/diplotene but instead quickly developed into probably NCOs in *Pdha2* ^*−/−*^ spermatocytes [2].

Overall, our analyses of the recombination process show that there appear to be normal DSBs and early recombination intermediates, but fewer mid/late recombination intermediates, and thus fewer COs. Therefore, PDHA2 is required to stabilise mid/late recombination intermediates for efficient CO formation.

### 3.4 PDHA2 Is Required for Efficient ATP and Acetyl‐CoA Production and Protein Acetylation in Germ Cells

**FIGURE 5:**
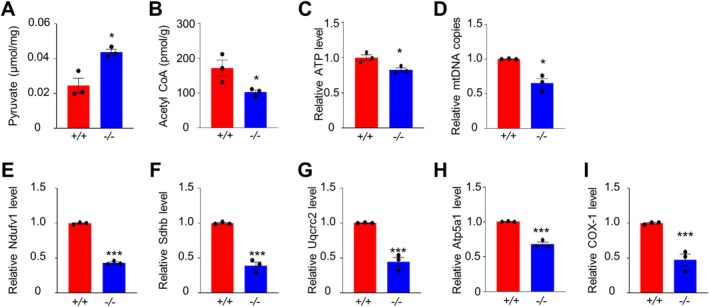
PDHA2 is required for the efficient generation of ATP and acetyl‐CoA in spermatocytes. (A) Increased pyruvate concentration in *Pdha2* ^*−/−*^ testes. (B and C) Decreased levels of acetyl‐CoA (B) and ATP (C) in *Pdha2* ^*−/−*^ testes. (D) Decreased mtDNA copy number in *Pdha2* ^*−/−*^ testes. (E–I) Decreased expression levels for five respiratory chain genes, Ndufv1 (E), Sdhb (F), Uqcrc2 (G), Atp5a1 (H) and COX‐1(I), in *Pdha2* ^*−/−*^ testes examined by RT‐qPCR. Error bar, SEM (*n* = 3 independent experiments). *p < 0.05; ***p < 0.001; two‐tailed *t*‐test.

PDHA2 is a component of the PDC, which catalyses the production of acetyl‐CoA from pyruvate. Acetyl‐CoA is then used as a substrate in the tricarboxylic acid cycle to generate ATP. As expected, pyruvate levels were significantly increased and acetyl‐CoA and ATP levels were significantly decreased in PD20 *Pdha2* ^*−/−*^ testes compared to WT (Figure 5A–C). Interestingly, the copy number of mtDNA and expression levels of representative components of respiratory chain‐related enzymes were all significantly decreased in the mutant (Figure 5D–I), suggesting that PDHA2 may also affect mitochondrial development and the expression of respiratory chain genes.

**FIGURE 6:**
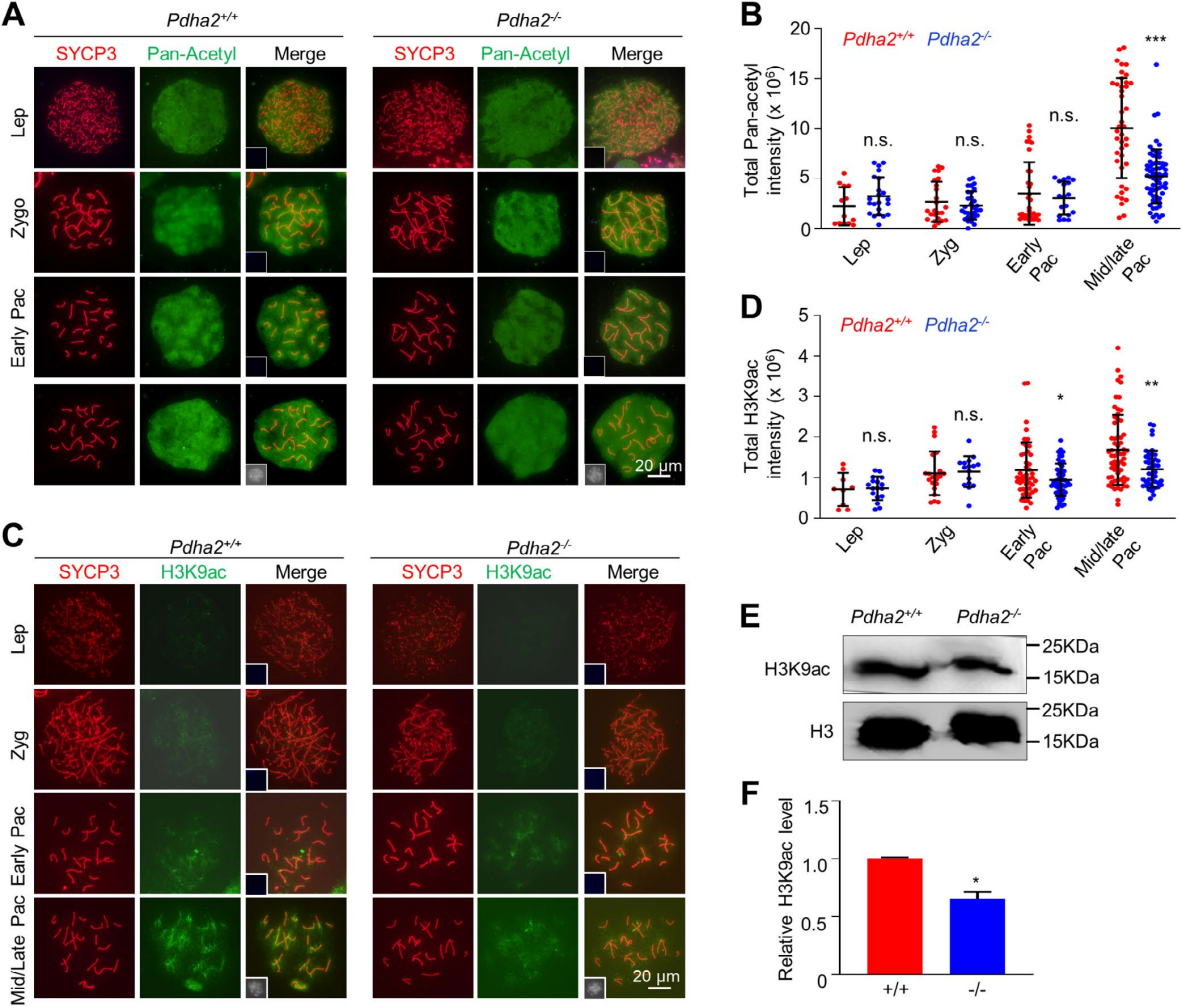
PDHA2 is required for efficient spermatocyte histone acetylation. (A and B) Representative images (A) and quantification (B) to show the acetylation level (pan‐acetyl, green) at different substages judged by SYCP3 signal (red) in surface spread spermatocytes. From left to right: *N* = 13, 22, 24, 31, 35, 18, 42, and 66 nuclei. (C and D) Representative images (C) and quantification (D) to show the H3K9ac level (green). From left to right: *N* = 9, 17, 21, 16, 46, 54, 62, and 42 nuclei. (E and F) Western blot (E) and quantification (F) of H3K9ac protein abundance from purified pachytene spermatocytes. Histone H3 was used as a loading control. Error bar, SD (B and D) or SEM (F). n.s. (not significant) *p* ≥ 0.05; *p < 0.05; **p < 0.01; ***p < 0.001; two‐tailed *t*‐test. Early Pac, early pachytene; Lep, leptotene; Mid/late Pac, middle/late pachytene; Zyg, zygotene.

Acetyl‐CoA also serves as a substrate for protein acetylation. Consistent with the decreased level of acetyl‐CoA in *Pdha2* ^*−/−*^ pachytene spermatocytes, the acetylation level was also significantly decreased when examined by immunostaining of spread spermatocytes with a pan acetylation antibody (Figure 6A,B). This result was further confirmed by the decreased H3K9Ac level when examined by immunostaining and western blot (Figure 6C–F). Modifications of acetylation, ubiquitination and methylation can compete for the same lysine residue. Along with decreased H3K9Ac, H3K9me3 and ubiquitylation (by FK2 antibody recognising mono‐ and poly‐ubiquitylation [54]) levels were significantly increased in the mutant pachytene spermatocytes (Figure S5A–H). We noted that the levels of these modifications in leptotene and zygotene spermatocytes were comparable between the mutant and WT (Figure 6C–F and Figure S5). This is consistent with the finding that PDHA2 is primarily expressed and functions from the pachytene. These results suggest the possibility that decreased acetyl‐CoA leads to decreased acetylation accompanied by increased methylation and ubiquitination modifications.

**FIGURE 7:**
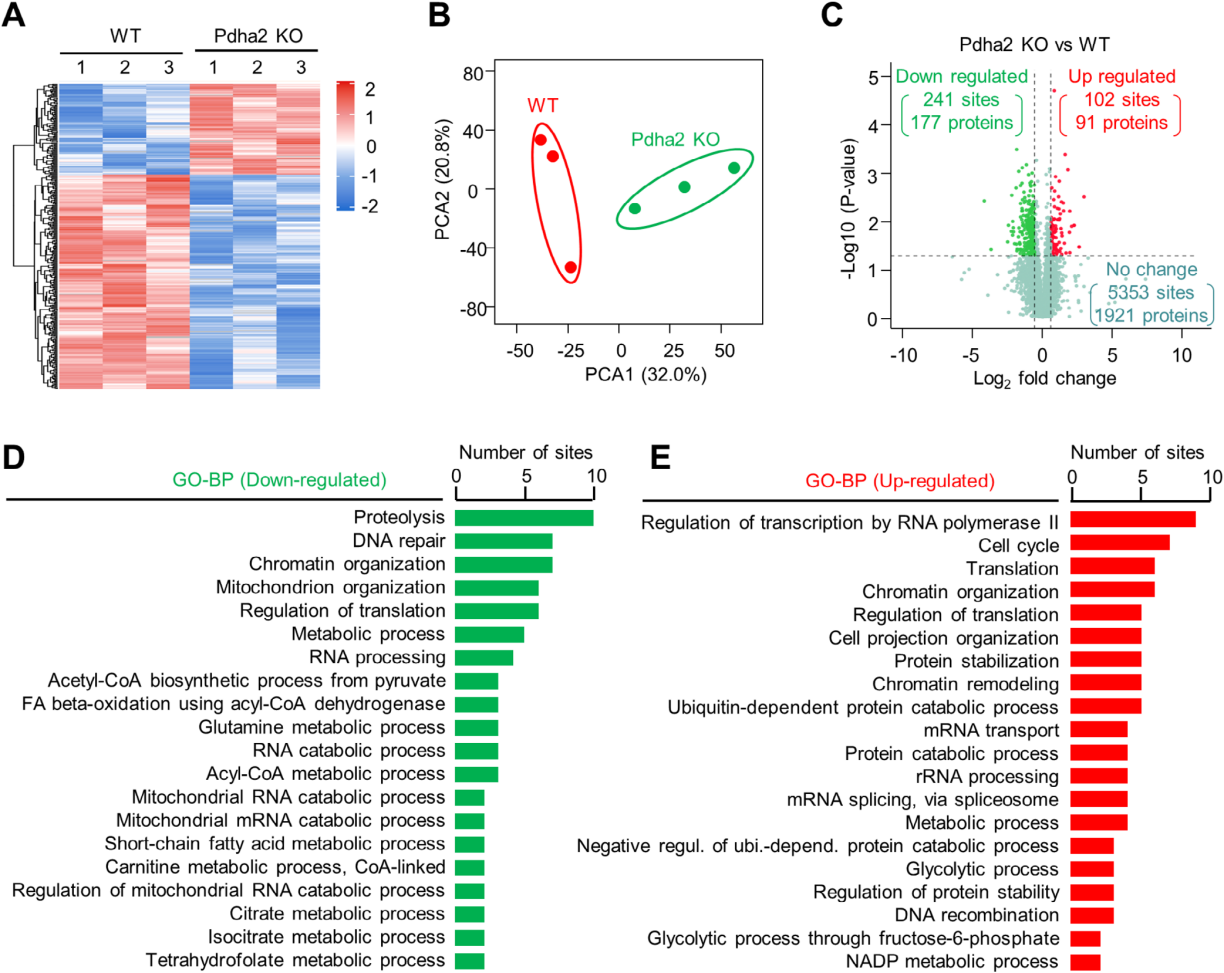
PDHA2 regulates spermatocyte acetylome. (A) Heatmap to show acetylation changes from pachytene spermatocyte acetylome. Colour key to show the relative level. (B) Principal component analysis (PCA) of acetylome. (C) Volcano plot to show up‐regulated (red) and down‐regulated (green) acetylation sites (|log2(fold change)| > 0.58 (1.5×) and adjusted *p* < 0.05). (D and E) GO analysis of down‐regulated (D) and up‐regulated acetylation of proteins (E).

To further characterise the global changes in acetylation modification, acetylated peptides from the WT and mutant pachytene spermatocytes were enriched, and label‐free quantitative proteomics was performed. A total of 5696 unique acetylation sites from 2189 proteins were identified. One hundred and two sites from 91 proteins showed a > 1.5‐fold increase, and 241 sites from 177 proteins showed a > 1.5‐fold decrease in acetylation (Figure 7A–C; Table S2), consisting with an overall decreased acetylation level. GO analysis showed that proteins with down‐regulated acetylation are mainly involved in proteolysis, DNA repair, chromatin organisation, mitochondrion organisation and translation (Figure 7D), and proteins with up‐regulated acetylation are mainly involved in transcription and translation, chromatin organisation/remodelling, cell cycle and protein stability/degradation (Figure 7E). The altered acetylation for proteins involved in various processes suggests the various roles of protein acetylation. Specifically, altered acetylation for proteins associated with mitochondria, chromatin organisation/remodelling and DNA repair suggests their possible roles in directly regulating these processes and is consistent with the observed mutant phenotypes (above). However, their actual roles require further investigation.

### 3.5 PDHA2 Regulates the Spermatocyte Transcription Profile

**FIGURE 8:**
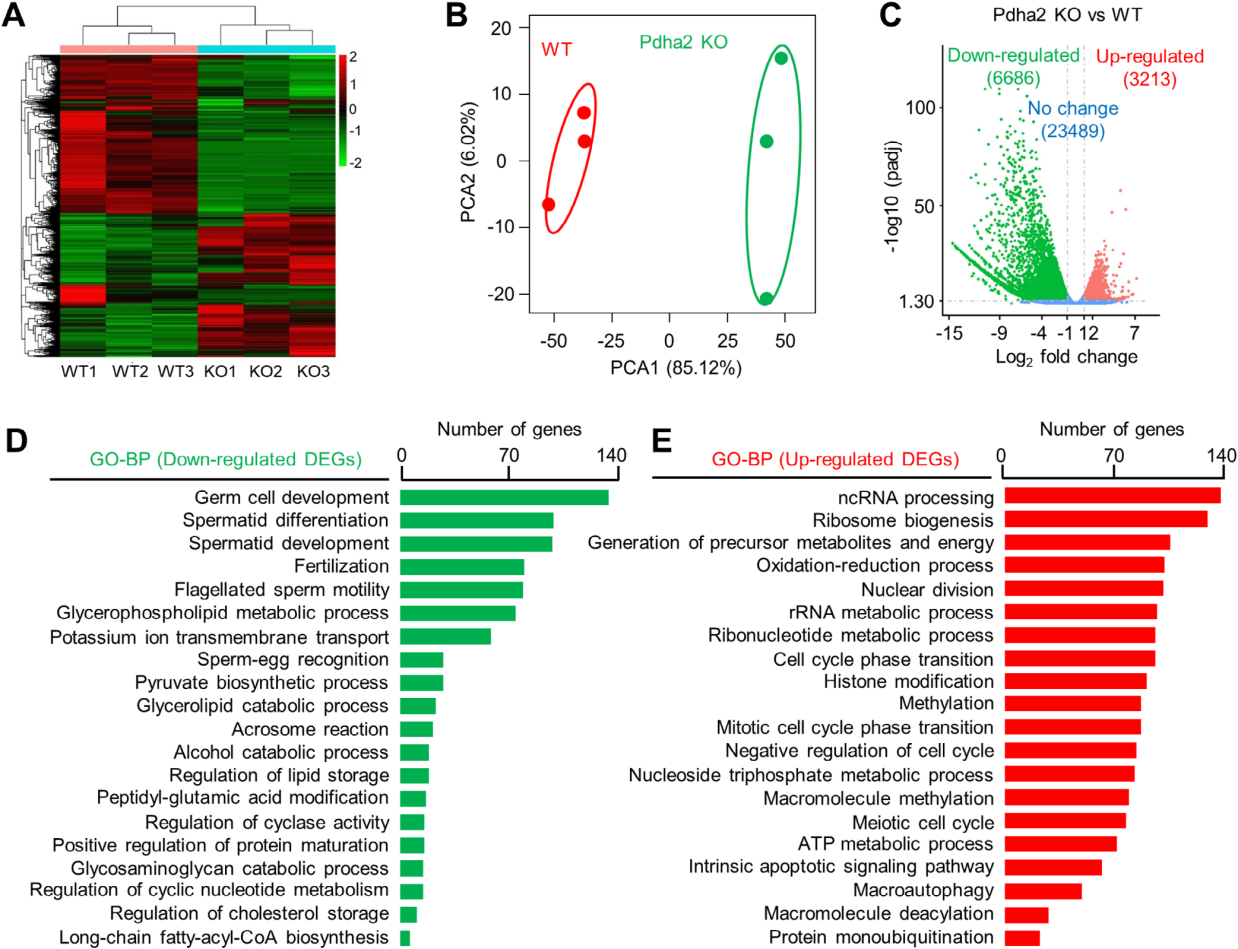
PDHA2 regulates spermatocyte transcriptome. (A) Heatmap to show the gene expression characteristics between different RNA‐seq data from pachytene spermatocytes. Colour key to show the relative gene expression level. (B) Principal component analysis (PCA) of RNA‐seq data. (C) Volcano plot to show up‐regulated (red) and down‐regulated (green) DEGs (TPM > 1, |log2(fold change)| > 1, and adjusted *p* < 0.05). (D) GO analysis of down‐regulated DEGs. (E) GO analysis of up‐regulated DEGs.

Since PDHA2 functions from pachytene (above), pachytene spermatocytes were collected from 8‐week‐old mice for RNA sequencing (RNA‐seq) to further investigate its role (Figure 8). Pachytene spermatocytes from the WT and mutant are comparable: (1) the mutant spermatocytes reach and pass through pachytene normally (also showing normal early and mid/late pachytene transition) (Figure 2A–C); (2) the XY body looks normal in the mutant (Figure S4A); (3) the expression of genes on the sex chromosomes is efficiently silenced at pachytene (below). Results from three independent experiments for each genotype showed good repeatability (Figure 8A,B; *R* ^2^ > 0.93 for repeats). Principal component analysis (PCA) revealed that *Pdha2* KO transcriptomes were clearly separated from WT (Figure 8B). RNA‐seq identified 6686 down‐regulated and 3213 up‐regulated DEGs (differentially expressed genes) among 33,388 detected genes (Figure 8C). The result that there were more down‐regulated DEGs is consistent with the decreased histone acetylation, as the level of histone acetylation is well correlated with transcriptional activity [55].

GO analysis of down‐regulated DEGs revealed that enriched processes were associated with the late spermatogenesis/sperm and ATP synthesis and metabolism, consistent with the pachytene/diplotene arrest and blocked conversion of pyruvate to acetyl‐CoA (Figure 8D). Interestingly, reduced expression levels of two axis components REC8 and STAG3 were also identified, and this result was further confirmed by RT‐qPCR, immunostaining of spread spermatocytes and western blot analysis of purified pachytene spermatocytes (Figure S6). Up‐regulated DEGs were enriched in several processes (Figure 8E). (1) Nuclear division and cell cycle: mainly the negative regulation of cell cycle progression and early/mid prophase of meiosis I, including meiotic DSB formation‐related genes (e.g., *Prdm9* and *Hormad1/2*). (2) Protein/DNA modification: mainly associated with up‐regulated methylation and deacetylation, consistent with decreased acetyl‐CoA and acetylation levels. (3) Nucleotide and energy metabolism. (4) Ribosome biogenesis and translation. The up‐regulation of genes in these two BPs probably reflects the feedback effect from a low acetyl‐CoA level caused shortage of precursors for metabolites and energy. (5) Apoptosis and autophagy. Thus, the altered gene expression profile is consistent with the functions of PDHA2 and the observed phenotypes. In the absence of PDHA2, there are fewer acetyl‐CoA and precursors for metabolites and energy. Consequently, the levels of acetylation (correspondingly increased methylation and ubiquitination), ATP and metabolites are decreased, which in turn promotes the expression of genes involved in these processes, ultimately leading to cell cycle progression defects and apoptosis.

### 3.6 PDHA2 Regulates Chromatin Accessibility of Spermatocytes

**FIGURE 9:**
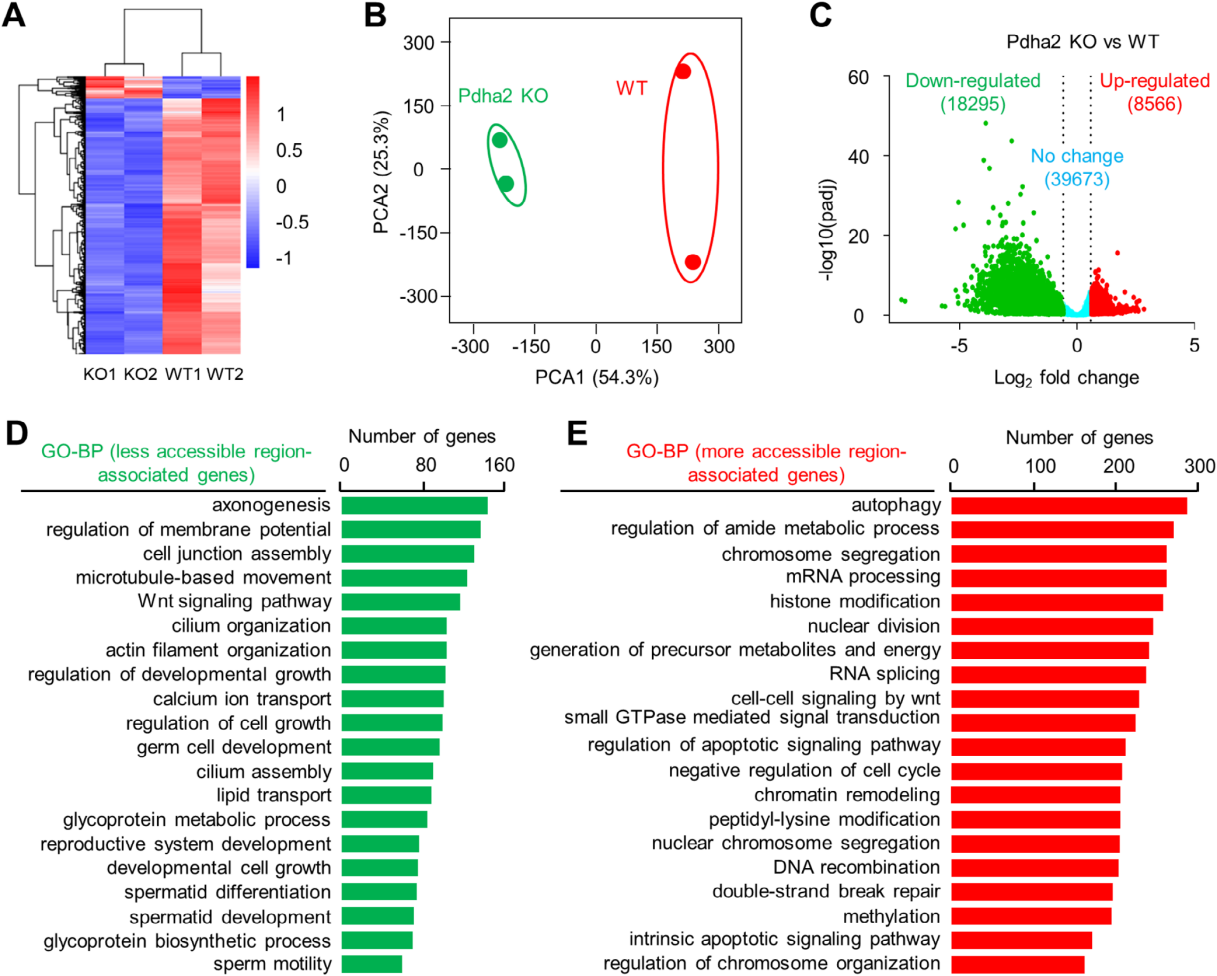
PDHA2 regulates chromatin accessibility of spermatocytes. (A) Heatmap to show chromatin accessibility between different ATAC‐seq data from pachytene spermatocytes. Colour key to show the relative levels of chromatin accessibility. (B) Principal component analysis (PCA) of ATAC‐seq data. (C) Volcano plot to show regions with increased (red) and decreased (green) chromatin accessibility (|log2 fold change| ≥ 1 and *p* < 0.05). (D and E) GO analysis of genes associated with decreased (D) and increased (E) chromatin accessibility.

Changes in transcription may result from altered histone acetylation induced changes in chromatin accessibility of spermatocytes. Thus, pachytene spermatocytes were collected from 8‐week‐old mice for ATAC‐seq (Figure 9). Results from two independent experiments for each genotype showed good repeatability (Figure 9A,B; *R* ^2^ > 0.94 for repeats). PCA revealed that chromatin accessibility from WT and *Pdha2* KO mice were well separated (Figure 9B). The analysis of ATAC‐seq data using DESeq2 identified 26,861 differentially accessible chromatin regions, including 18,295 decreased and 8566 increased chromatin accessible regions in the mutant (Figure 9C). This result of more regions with decreased chromatin accessibility is consistent with the observation of more genes with decreased expression and decreased histone acetylation.

**FIGURE 10:**
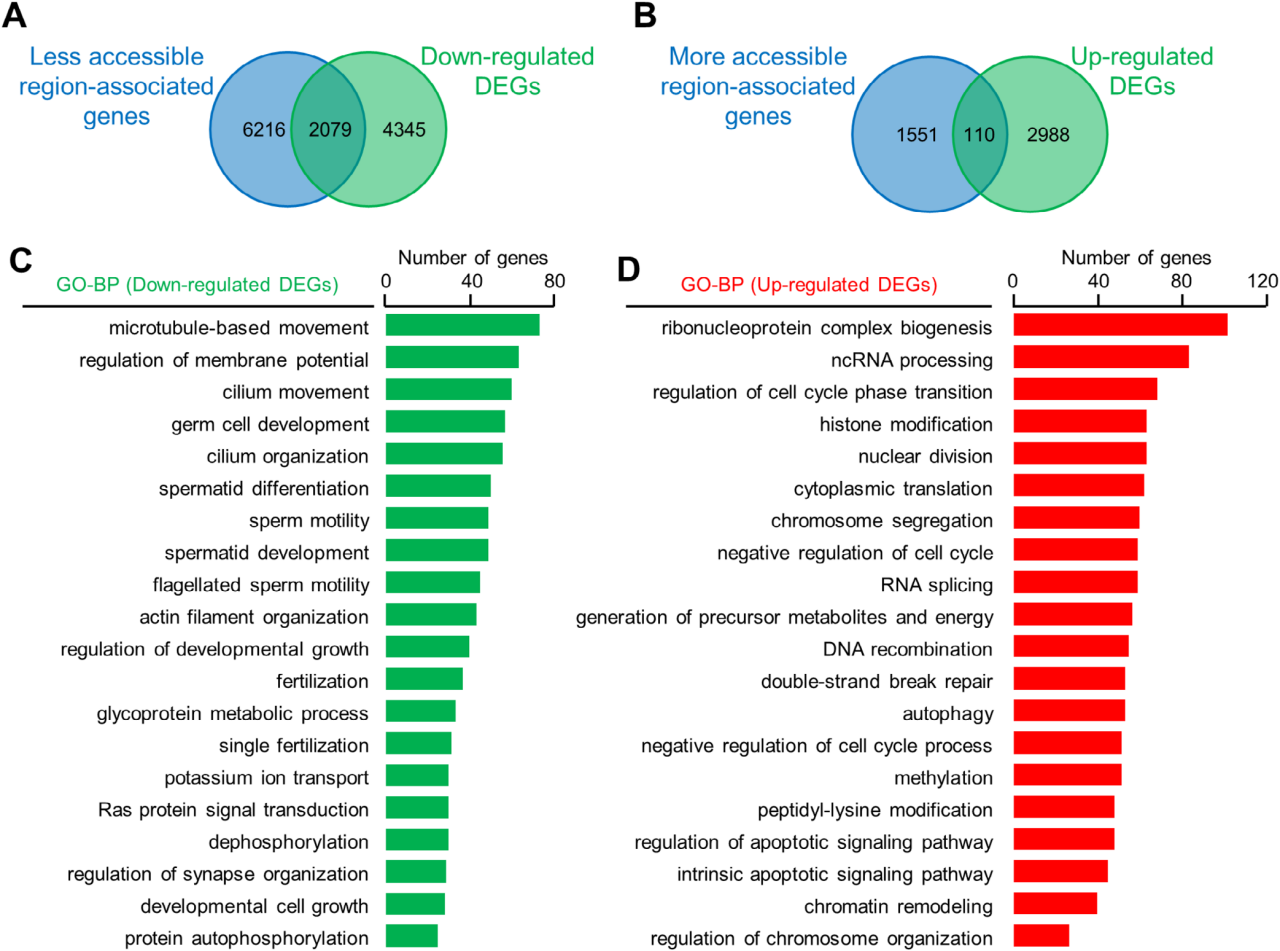
Integration analysis of ATAC‐seq and RNA‐seq data. (A) Venn plot to show the overlap between less accessible chromatin region‐associated genes from ATAC‐seq and down‐regulated genes from RNA‐seq. (B) Venn plot to show the overlap between more accessible chromatin region‐associated genes from ATAC‐seq and up‐regulated genes from RNA‐seq. (C) GO enrichment analysis of overlapped genes in (A). (D) GO enrichment analysis of overlapped genes in (B).

Peak annotation using BEDTools showed that regions with decreased and increased chromatin accessibility were associated with 8295 and 1661 genes, respectively. GO analysis revealed that genes associated with decreased chromatin accessibility were primarily involved in the processes of late spermatogenesis/sperm and ATP synthesis and metabolism. Genes associated with increased chromatin accessibility were enriched in processes related to apoptosis and autophagy, nuclear division and cell cycle, protein/DNA modification, nucleotide and energy metabolism and ribosome biogenesis and translation (Figure 9D,E). Further integration analysis of ATAC‐seq (associated genes) and RNA‐seq data showed that ~1/3 of down‐regulated DEGs could be interpreted with more compacted chromatin and only a few up‐regulated DEGs could be interpreted with more open chromatin (Figure 10A,B). GO analysis of DEGs associated with altered chromatin accessibility showed that they are involved in the same BPs as revealed by RNA‐seq and ATAC‐seq experiments (Figure 10C,D). These findings indicate that PDHA2 regulates histone acetylation, thereby modulating chromatin accessibility and the transcriptome of spermatocytes.

## 4 Discussion

We showed that *Pdha2* KO spermatocytes had defective chromosome structure and decreased levels of mid/late recombination intermediates and COs, although the early stage of the meiotic recombination process appeared normal. As a result, *Pdha2* KO mice were male infertile.

### 4.1 PDHA2 Is Required to Stabilise Recombination Intermediates and Thus Efficient CO Formation

In the absence of PDHA2, meiotic DSBs appear to form timely and efficiently, and the levels of early recombination intermediates (as indicated by foci of RPA, RAD51, DMC1, etc.) are not affected. Consistently, no defects in homologue synapsis are observed. However, the level of mid/late recombination intermediates (as indicated by foci of MSH4, ZZS and RNF212) is significantly decreased from early pachytene. As a result, the level of COs is slightly but significantly decreased, with more chromosomes losing the obligatory CO.

Interestingly, it appears that all DSBs are repaired, as indicated by the immunostaining results of γH2AX, RPA, RAD51 and DMC1 at pachytene. In these diplotene‐like spermatocytes, γH2AX signal reappears on autosomes, probably indicating that additional DSBs or apoptosis occur in these nuclei. These results suggest that the mid/late recombination intermediates are less stabilised, probably due to the impaired recombination proteins (e.g., due to altered acetylation), decreased ATP level, or defective chromosome structure in the absence of PDHA2 (see below). Thus, a fraction of them should be developed into COs, but instead are rapidly developed into NCOs or repaired with sister chromatids as templates at pachytene in *Pdha2* ^*−/−*^ spermatocytes [2].

### 4.2 PDHA2 Regulates Chromosome Structure/Organisation

Our analysis of meiotic progression shows that *Pdha2* KO spermatocytes can timely progress to pachytene, and the proportion of early (H1t‐negative) versus mid/late (H1t‐positive) pachytene spermatocytes is comparable to that in WT. Interestingly, however, a fraction of *Pdha2* KO spermatocytes shows a unique ‘diplotene‐like’ morphology: fragmented chromosome axes not observed in other reported mutants, with some regions of chromosomes losing both SYCP3 (an axis component) and SYCP1 (a central SC component) signal. The early loss of SYCP3 indicates a defective chromosome structure in *Pdha2* KO spermatocytes.

The proposal that PDHA2 is required for proper meiotic chromosome structure/organisation is further supported by several observations in this mutant. (1) *Pdha2* KO pachytene spermatocytes show decreased histone acetylation but increased methylation and ubiquitination levels, which play an important role in chromosome organisation. (2) OA treatment failed to induce *Pdha2* KO pachytene spermatocytes to progress to metaphase I, suggesting that these spermatocytes are at an inappropriate chromatin status. (3) Defective chromosome ends are revealed by fewer TRF1 foci in both pachytene and diplotene‐like spermatocytes. (4) The abundance of axis components REC8 and STAG3 on chromosomes was reduced. (5) The reappearance of γH2AX signal on autosomes, probably indicating altered chromosome structure, allows the reoccurrence of additional DSBs in these diplotene‐like spermatocytes, similar to *Zfp541* mutants [33, 56]. (6) ATAC‐seq analysis indicates the chromatin accessibility is significantly changed in *Pdha2* KO mutants. (7) A more diffuse DAPI signal for pachytene spermatocytes is observed in *Pdha2* KO mutants. These results suggest that chromosome structure defects in *Pdha2* KO are evident at least from the pachytene stage.

An important question is how PDHA2 regulates chromosome structure/organisation. One obvious possibility is that PDHA2 affects the acetyl‐CoA level, which regulates acetylation of histones and other proteins, and consequently other types of modifications, as observed. Decreased histone acetylation would contribute to more compacted chromatin as revealed by the overall reduced chromatin accessibility (from ATAC‐seq) and gene expression level (from RNA‐seq) [57, 58]. It is also possible that the altered abundance of some chromatin organisation‐related critical factors in *Pdha2* KO spermatocytes impairs the chromosome structure, for example, decreased abundance of the cohesin components REC8 and STAG3 on chromosomes would destabilise chromosome axes [59]. Interestingly, ATP is required for cohesin loading, and RAD21L‐cohesin is replaced by RAD21‐cohesin from pachytene [60, 61, 62], thus, a lower level of ATP may affect the loading of RAD21‐cohesin. Moreover, decreased levels of ATP and acetyl‐CoA would result in decreased SMC3 acetylation and early release of cohesin from chromosomes in *Pdha2* KO spermatocytes [60, 63].

Since chromosome organisation and recombination are tightly linked at both the DNA and structural levels [2], the above discussion further raises the intriguing question of whether recombination defects are actually caused by the defective chromosome structure. In the absence of PDHA2, the decreased level of mid/late recombination intermediates at pachytene could be caused by the decreased level of ATP, since the recombination process requires ATP, for example, MSH4/5 requires ATP to function properly [53]. Consistently, mutants with defective mitochondrial structure/function and thus decreased ATP show spermatogenesis arrest during the recombination process [64]. Since both chromosome structure and meiotic recombination defects are observed from early pachytene, it is also very reasonable that defective chromosome organisation, for example, precocious removal of cohesins disturbing chromosome axes, impairs recombination. It is certainly possible that the decreased ATP level, together with altered chromosome structure, destabilises mid/late recombination intermediates and thus ultimately impairs CO formation.

### 4.3 A Testis‐Specific PDHA2 Substitutes an X‐Linked PDHA1 From Pachytene During Spermatogenesis

The X and Y chromosomes are transcriptionally suppressed by meiotic sex chromosome inactivation (MSCI) and are sequestered in the sex body from pachytene in male meiosis [65, 66]. Many genes on the sex chromosomes have corresponding retrotransposed paralogs on autosomes, and at least some retrogenes have important functions during male meiosis, either to compensate the function of the silenced gene or obtain modified functions (e.g., [40, 66, 67]).

Our current finding, in combination with previous reports, reveals that the testis‐specific PDHA2 substitutes its X‐linked paralog PDHA1 from pachytene during spermatogenesis. (1) PDHA2 is specifically expressed in testes mainly from pachytene, however, PDHA1 is expressed only before pachytene in male meiosis and in other cells (Figure 1) [23, 24, 25, 26, 30]. Consistent with their expression patterns, *Pdha2* ^*−/−*^ male mice show meiotic defects from pachytene (Figures 2, 3, 4, 5, 6). (2) Functionally, PDHA2 substitutes PDHA1 as a core subunit of PDC and is required for the conversion of pyruvate to acetyl‐CoA (Figures 5 and 6). Interestingly, a higher abundance of all subunits of the PDC complex, including PDHA2, is observed from pachytene throughout meiosis [30], indicating that more acetyl‐CoA and ATP may be required during this period. Consistently, a higher level of protein acetylation is also observed in pachytene spermatocytes (Figure 6). To better understand the relationship between PDHA1 and PDHA2, it is also worth examining whether PDHA2 has enhanced enzyme activity, whether forced/ectopic expression of PDHA2 can functionally substitute PDHA1 in somatic cells, and whether PDHA1 can substitute PDHA2 during meiosis.

## Author Contributions

G.W., L.Z. and S.W. conceived and designed the experiments. C.D.C. and K.F. provided the knockout mice. G.W., Y.S., X.Z., Q.S., S.L., P.W. and X.Z. executed the experiments. G.W., L.Z. and S.W. analysed the data and wrote the manuscript with inputs and edits from all authors.

## Conflicts of Interest

The authors declare no conflicts of interest.

## Supporting information


Data S1.

Table S2.

## Data Availability

RNA‐seq and ATAC‐seq data have been deposited at NCBI SRA under the accession numbers PRJNA1047259 and PRJNA1127797, respectively. Other data that support the findings of this study are available from the corresponding author upon reasonable request.
