# Supplementary material for: Testis‐Specific PDHA2 Is Required for Proper Meiotic Recombination and Chromosome Organisation During Spermatogenesis
Source: Cell Prolif. 2025 Feb 20;58(7):e70003. doi: 10.1111/cpr.70003 (PMC12240641; doi:10.1111/cpr.70003)
Supplement: Supplementary file 1 — Data S1. [file CPR-58-e70003-s002.pdf]

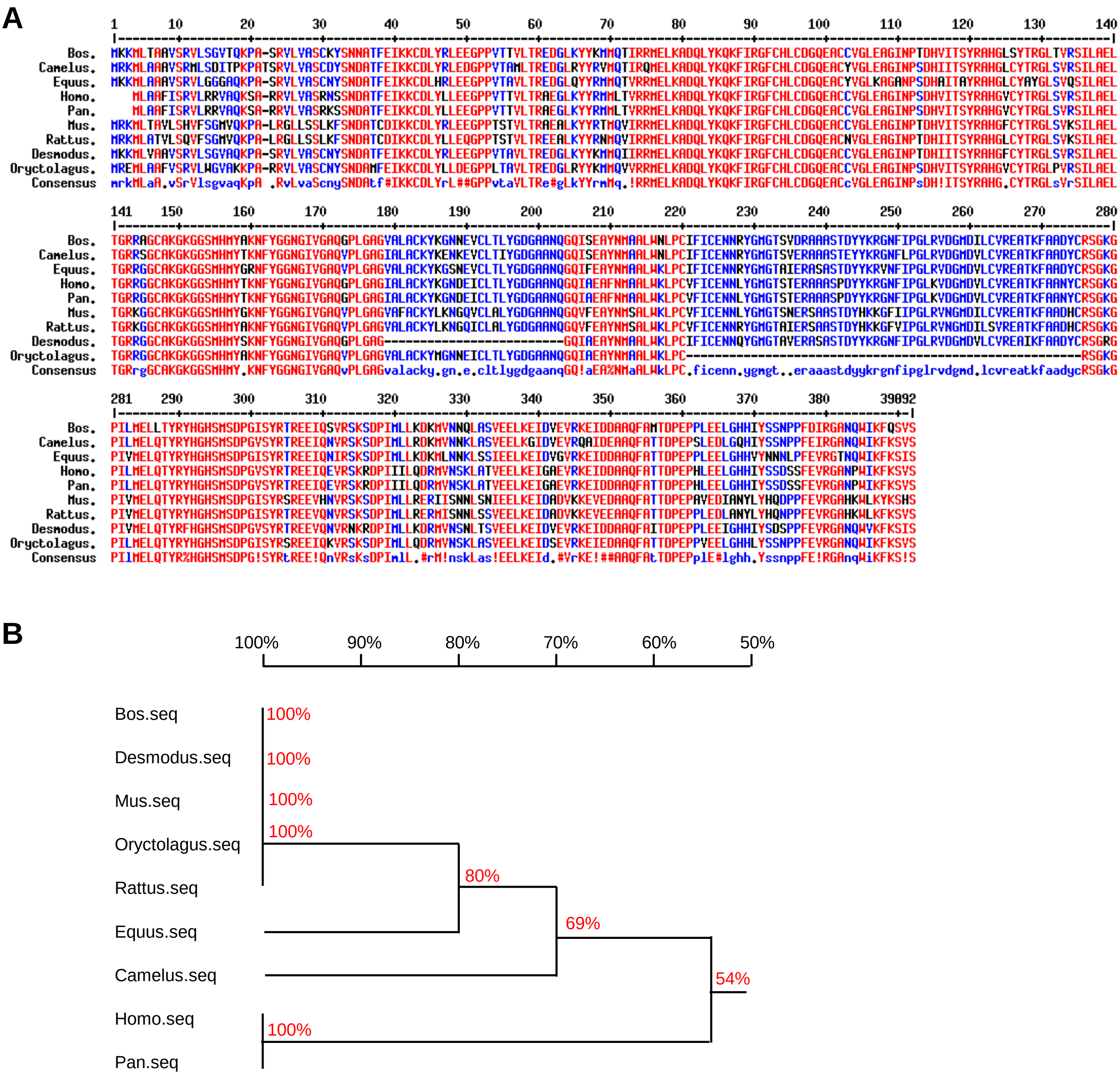

Figure S1. PDHA2 is highly conserved in vertebrates.

(A) PDHA2 protein sequences were downloaded from the NCBI protein database and the alignment was performed using Multalin (http://multalin.toulouse.inra.fr/multalin/). **(B)** Phylogenetic tree of PDHA2 was drawn by DNAMAN software. Percentages indicate evolutionary distances. Bos, *Bos taurus*; Camelus, *Camelus ferus*; Equus, *Equus caballus*; Homo, *Homo sapiens*; Pan, *Pan troglodytes*; Mus, *Mus musculus*; Rattus, *Rattus norvegicus*; Desmodus, *Desmodus rotundus*; Oryctolagus, *Oryctolagus cuniculus*.

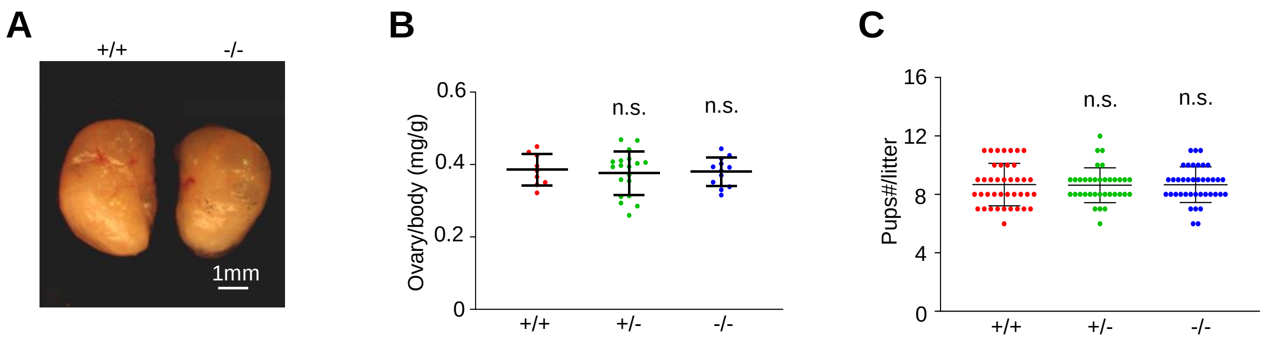

Figure S2. PDHA2 is not required for female fertility.

(A) Representative images for ovaries from 8-week-old mice. **(B)** Quantification of ratios of ovary/body weight. Each dot represents one mouse. n = 9 (WT, +/+), 19 (heterozygote, +/-), and 12 (knockout, -/-) female mice. **(C)** The number of pups per litter. From left to right, n=40, 35, and 39 litters (6 female mice for each genotype). Error bar, SD (B, C); n.s. (not significant), p>=0.05; two-tailed t-test.

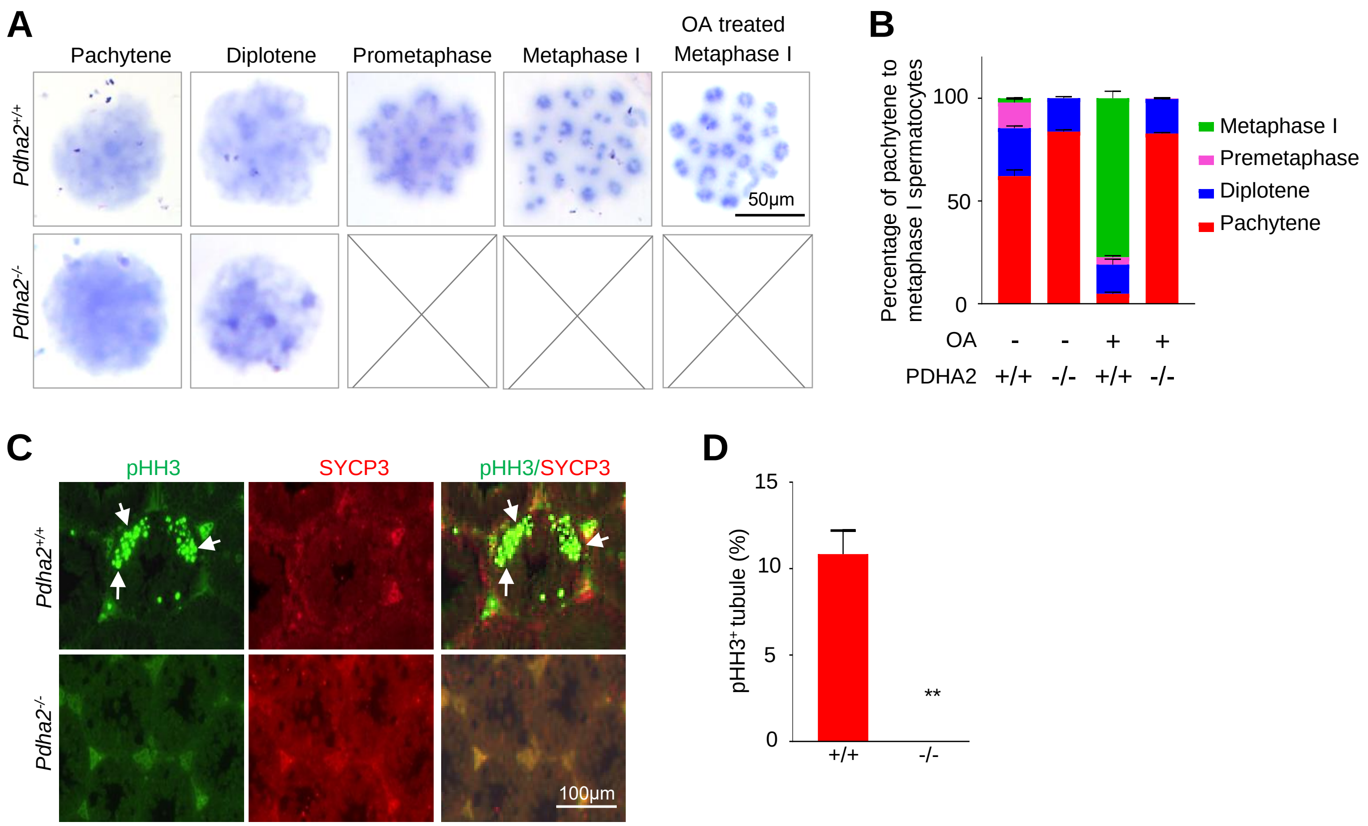

Figure S3. PDHA2 is required for normal meiotic progression.

(A,B) Representative images (A) and quantification (B) of spermatocytes from pachytene to metaphase I by Giemsa staining. For OA treatment, spermatocytes were cultured in 4 μM OA-supplemented α-MEM medium for 5h. Chi-square test. Error bar, 95% confidence interval. From left to right: n= 798, 523, 512, and 539 pachytene to metaphase I spermatocytes. **(C,D)** Immunostaining (C) and quantification (D) of phosphorylated histone H3 serine 10 (pHH3) in 8-week-old testis sections. White arrows, pHH3 signal. Error bar, SEM (n=3 independent experiments); **, $p < 0.01$; two-tailed t-test.

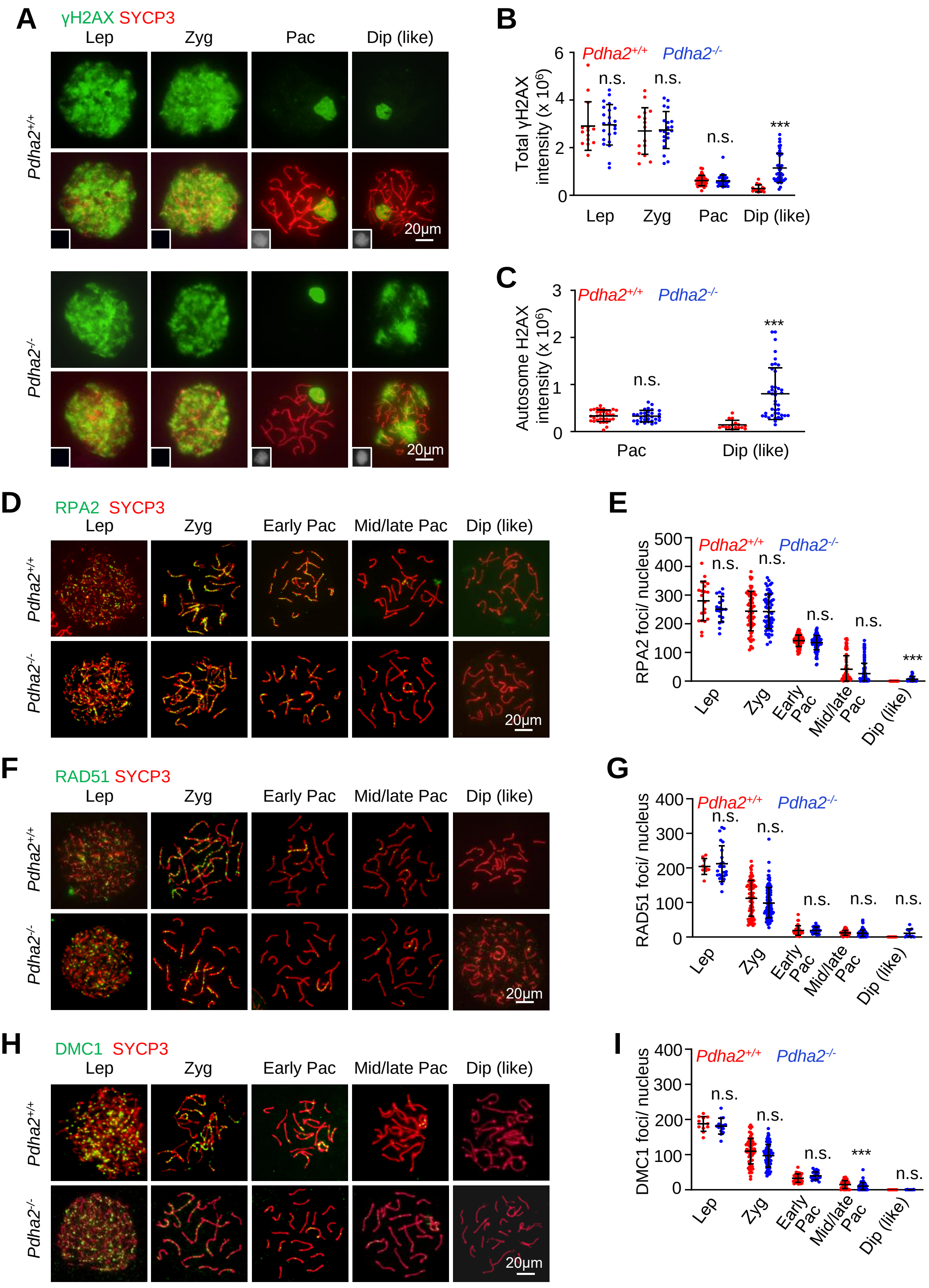

A
γH2AX SYCP3
Lep
Zyg
Pac
Dip (like)
Pdha2+/+
Pdha2-/-
20μm
B
Pdha2+/+ Pdha2-/-
Total γH2AX intensity (x 10^6)
n.s.
***
Lep
Zyg
Pac
Dip (like)
C
Autosome H2AX intensity (x 10^6)
D
RPA2 SYCP3
Early Pac
Mid/late Pac
E
RPA2 foci/ nucleus
F
RAD51 SYCP3
G
RAD51 foci/ nucleus
H
DMC1 SYCP3
I
DMC1 foci/ nucleus

Figure S4. PDHA2 is not required for DSB formation.

(A) Representative images of γH2AX (green) in surface spread spermatocytes. Stages were determined based on SYCP3 (red) and H1t (grey). **(B,C)** Quantification of total (B) and autosome (C) γH2AX signal intensity per nucleus. From left to right: n = 14, 23, 15, 20, 33, 31, 16, and 41 nuclei (B), 33, 31, 16, and 41 nuclei (C). Nuclei measured in (C) are the same set of nuclei measured in (B). **(D-I)** Representative images of RPA2 (D), RAD51 (F), and DMC1 (H) in surface spread spermatocytes and quantification of RPA2 (E), RAD51 (G), and DMC1 (I) foci. From left to right: n = 21, 18, 67, 58, 92, 95, 84,157, 40, and 20 nuclei (E); 9, 24, 71, 115, 35, 28, 70, 78, 24, and 12 nuclei (G); 11, 14, 69, 78, 34, 22, 40, 57, 23, and 18 nuclei (I). Error bar, SD (B,C,E,G,I). n.s. (not significant), $p>=0.05$; ***, $p < 0.001$; two-tailed t-test. Lep, Leptotene; Zyg, Zygotene; Early Pac, Early Pachytene; Mid/late Pac, Middle/late Pachytene; Dip, Diplotene; Dip-like, Diplotene-like.

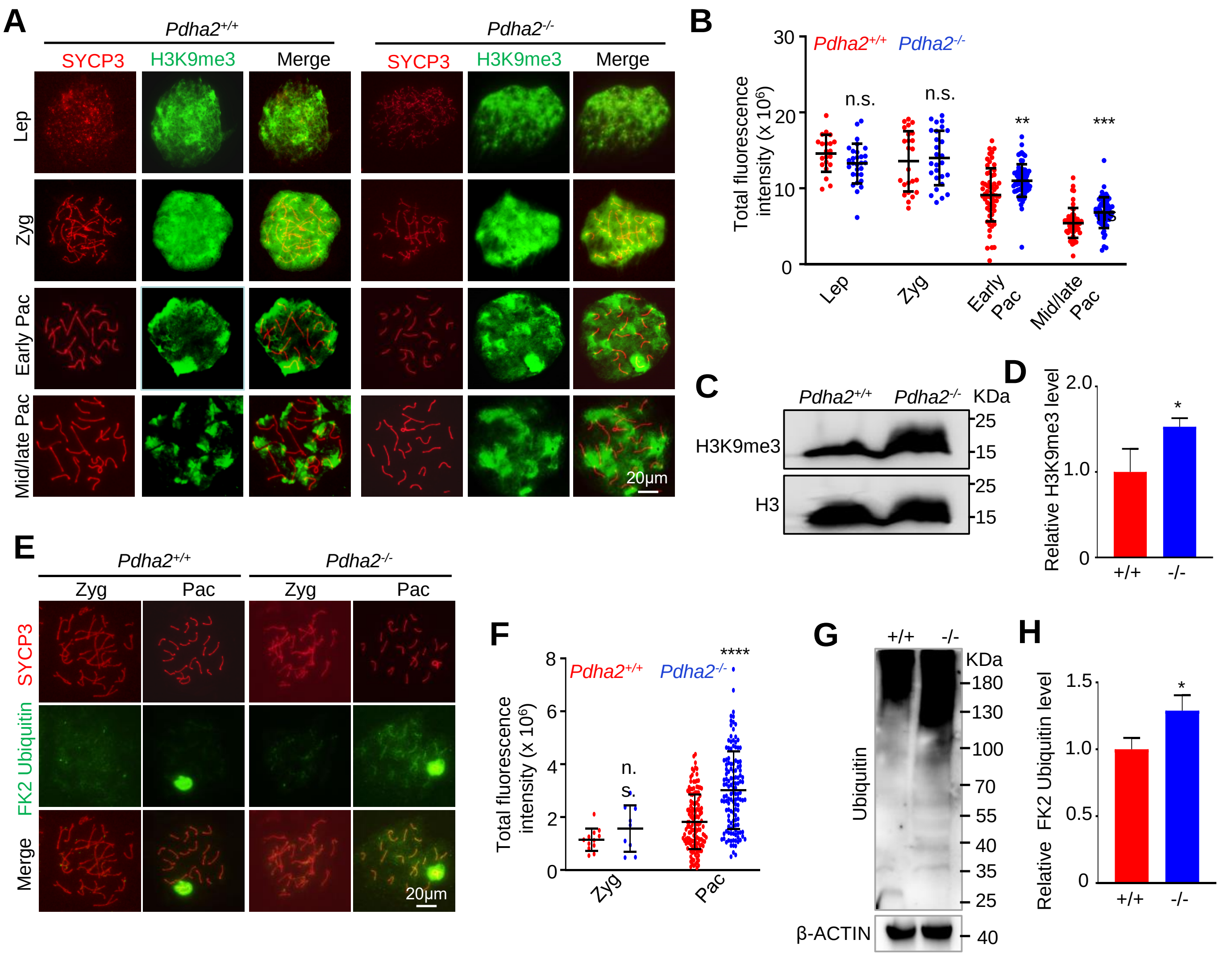

Figure S5. PDHA2 deficiency increases H3K9me3 and Ubiquitin levels in spermatocytes.

(A,B) Representative images (A) and quantification (B) of H3K9me3 (green) in surface spread spermatocytes. From left to right: n= 21, 29, 24, 27, 60, 74, 54, and 58 nuclei. (**C,D**) Western blot (C) and quantification (D) of H3K9me3 protein abundance from purified pachytene spermatocytes. Histone H3 was used as a loading control. n=3 independent experiments. **(E,F)** Representative images (E) and quantification (F) of ubiquitination level (green) in surface spread spermatocytes. The FK2 anti-ubiquitin antibody recognizes mono- and poly-ubiquitylated proteins. From left to right: n= 12, 9,140, and 130 nuclei. **(G,H)** Western blot (G) and quantification (H) of the ubiquitination level from purified pachytene spermatocytes. n=3 independent experiments. β-ACTIN was used as a loading control. Error bar, SD (B, F) or SEM (D, H). n.s. (not significant), $p>=0.05$; *, $p<0.05$; **, $p<0.01$; ***, $p<0.001$; two-tailed t-test. Lep, leptotene; Zyg, zygotene; Early Pac, Early Pachytene; Mid/late Pac, Middle/late Pachytene; Pac-like, Pachytene-like.

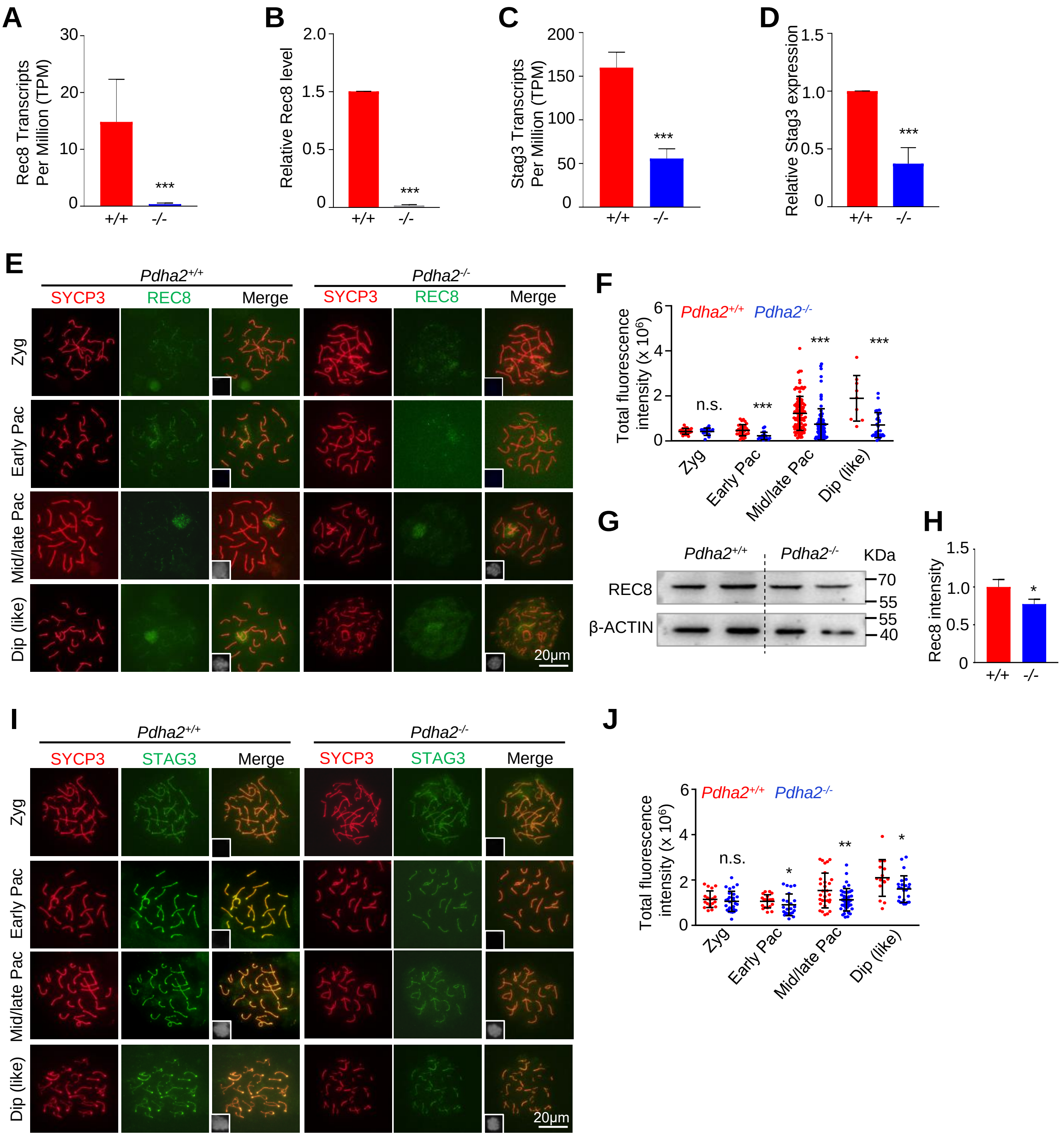
A
Rec8 Transcripts Per Million (TPM)
30
20
10
0
***
+/+
-/-
B
Relative Rec8 level
2.0
1.5
0.5
0
***
+/+
-/-
C
Stag3 Transcripts Per Million (TPM)
200
150
100
50
0
***
+/+
-/-
D
Relative Stag3 expression
1.5
1.0
0.5
0
***
+/+
-/-
E
Pdha2+/+
Pdha2-/-
SYCP3
REC8
Merge
Zyg
Early Pac
Mid/late Pac
Dip (like)
20µm
F
Total fluorescence intensity (x 10^6)
Pdha2+/+
Pdha2-/-
n.s.
***
Zyg
Early Pac
Mid/late Pac
Dip (like)
G
KDa
REC8
β-ACTIN
70
55
55
40
H
Rec8 intensity
1.5
1.0
0.5
0
*
+/+
-/-
I
Pdha2+/+
Pdha2-/-
SYCP3
STAG3
Merge
Zyg
Early Pac
Mid/late Pac
Dip (like)
20µm
J
Total fluorescence intensity (x 10^6)
Pdha2+/+
Pdha2-/-
n.s.
*
**
Zyg
Early Pac
Mid/late Pac
Dip (like)

Figure S6. Decreased REC8 and STAG3 abundance in PDHA2 deficient spermatocytes.

(**A, B**) Relative Rec8 mRNA levels observed from RNA-seq (A) and RT-qPCR (B). (**C, D**) Relative Stag3 mRNA levels observed from RNA-seq (C) and RT-qPCR (D). Error bar, SEM; n = 3 independent experiments. ***, p<0.001; Student's t-test. **(E,F)** Representative images (E) and quantification (F) of REC8 (green) in surface spread spermatocytes. Zyg, zygotene; Early Pac, Early Pachytene; Mid/late Pac, Middle/late Pachytene; Dip, Diplotene; Dip-like, Diplotene-like. From left to right: n= 22, 15, 31, 19, 109, 94, 9, and 30 nuclei. Error bar, SD. n.s. (not significant), p>=0.05; ***, p<0.001; two-tailed t-test. **(G,H)** Western blot (G) and quantification (H) of REC8 abundance from purified pachytene spermatocytes. Error bar, SEM; n = 3 independent experiments. **, p<0.01; Student's t-test. **(I,J)** Representative images (I) and quantification (J) of STAG3 (green) in surface spread spermatocytes. Zyg, zygotene; Early Pac, Early Pachytene; Mid/late Pac, Middle/late Pachytene; Dip, Diplotene; Dip-like, Diplotene-like. From left to right: n= 18, 28, 20, 21, 30, 43, 15, and 24 nuclei. Error bar, SD. n.s. (not significant), p>=0.05; *, p<0.05; **, p<0.01; two-tailed t-test. We noticed the dramatic decrease of Rec8 and Stage 3 mRNA vs moderately decreased proteins, which may indicate the feedback regulation at the translation level.

Table S1. Primers used in this study.

Primers	Sequence (5'-3')	Purpose
Pdha2-exon1-Sg	GGAGGCAGGGATAAATCCCACGG	SgRNA for Pdha2 KO
Pdha2-Forward	AAAGTGTGGGAGATCCTCCC	WT and mutant genotyping
Pdha2-WT-Reverse	CCGTGGGATTTATCCCTGCC	WT genotyping (with Pdha2-Forward)
Pdha2-Mutant-Reverse	ACGTGATCCGTGCCTCCAGC	Mutant genotyping (with Pdha2-Forward)
mPdha2-Forward	GGCTATGTCCCTACCCTG	*Pdha2* expression
mPdha2-Reverse	GCCTGCTCCTCTGATGCT	
β-actin-Forward	CATCCGTAAAGACCTCTATGCCAAC	*β-Actin* expression (control)
β-actin-Reverse	ATGGAGCCACCGATCCACA	
Atp5a1-Forward	TCTCCATGCCTCTAACACTCG	*Atp5a1* expression
Atp5a1-Reverse	CCAGGTCAACAGACGTGTCAG	
Ndufv1-Forward	TTTCTCGGCGGGTTGGTTC	*Ndufv1* expression
Ndufv1-Reverse	GGTTGGTAAAGATCCGGTCTTC	
Sdhb-Forward	AATTTGCCATTTACCGATGGGA	*Sdhb1* expression
Sdhb-Reverse	AGCATCCAACACCATAGGTCC	
Uqcrc2-Forward	AAAGTTGCCCCGAAGGTTAAA	*Uqcrc2* expression
Uqcrc2-Reverse	GAGCATAGTTTTCCAGAGAAGCA	
COX-1-Forward	GTGCTGGGGCAGTGCTGGAG	*COX-1* expression
COX-1-Reverse	TGGGGCCTGAGTAGCCCGTG	
Mt16S-Forward	CCGCAAGGGAAAGATGAAAGAC	*Mt16S* copy number
Mt16S-Reverse	TCGTTTGGTTTCGGGGTTTC	
ApoB-Forward	CGTGGGCTCCAGCATTCTA	*APoB* copy number (control)
ApoB-Reverse	TCACCAGTCATTTCTGCCTTTG	
Rec8-Forward	TAGTGTGCTGGTAAGAGTGCAAC	*Rec8* expression
Rec8-Reverse	TGTCTTCCACAAGGTACTGGC	
Stag3-Forward	TCCTCAGGCAGTGAGTCTTCC	*Stag3* expression
Stag3-Reverse	GTTCCCTGTGAGTCTCTGTCAT	

Table S2. Identified acetylation sites and peptides from pachytene spermatocytes.
